# Offline ensemble co-reactivation links memories across days

**DOI:** 10.1038/s41586-024-08168-4

**Published:** 2024-11-06

**Authors:** Yosif Zaki, Zachary T. Pennington, Denisse Morales-Rodriguez, Madeline E. Bacon, BumJin Ko, Taylor R. Francisco, Alexa R. LaBanca, Patlapa Sompolpong, Zhe Dong, Sophia Lamsifer, Hung-Tu Chen, Simón Carrillo Segura, Zoé Christenson Wick, Alcino J. Silva, Kanaka Rajan, Matthijs van der Meer, André Fenton, Tristan Shuman, Denise J. Cai

**Affiliations:** 1 Nash Department of Neuroscience, Icahn School of Medicine at Mount Sinai, New York, NY USA; 2 Department of Psychological & Brain Sciences, Dartmouth College, Hanover, NH USA; 3 Graduate Program in Mechanical and Aerospace Engineering, Tandon School of Engineering, New York University, Brooklyn, NY USA; 4 Department of Neurobiology, Psychiatry & Biobehavioral Sciences and Psychology, Integrative Center for Learning and Memory, Brain Research Institute, University of California, Los Angeles, Los Angeles, CA USA; 5 Center for Neural Science, New York University, New York, NY USA; 6 Neuroscience Institute at the NYU Langone Medical Center, New York, NY USA

**Keywords:** Hippocampus, Neural circuits, Consolidation

## Abstract

Memories are encoded in neural ensembles during learning^1–6^ and are stabilized by post-learning reactivation^7–17^. Integrating recent experiences into existing memories ensures that memories contain the most recently available information, but how the brain accomplishes this critical process remains unclear. Here we show that in mice, a strong aversive experience drives offline ensemble reactivation of not only the recent aversive memory but also a neutral memory formed 2 days before, linking fear of the recent aversive memory to the previous neutral memory. Fear specifically links retrospectively, but not prospectively, to neutral memories across days. Consistent with previous studies, we find that the recent aversive memory ensemble is reactivated during the offline period after learning. However, a strong aversive experience also increases co-reactivation of the aversive and neutral memory ensembles during the offline period. Ensemble co-reactivation occurs more during wake than during sleep. Finally, the expression of fear in the neutral context is associated with reactivation of the shared ensemble between the aversive and neutral memories. Collectively, these results demonstrate that offline ensemble co-reactivation is a neural mechanism by which memories are integrated across days.

## Abstract (web summary)

In mice, a strong aversive experience drives offline ensemble reactivation of not only the recent aversive memory but also a neutral memory formed 2 days before, linking fear of the recent aversive memory to the previous neutral memory.

## Main

Individual memories are initially encoded by ensembles of cells that are active during a learning event^1–6^ and are stabilized during offline periods after learning through reactivation of those ensembles^7–17^. These reactivations often occur in brief synchronous bursts, which are necessary to drive memory consolidation^13,18–21^. Most research on episodic memory has focused on how the brain maintains stable representations of discrete memories; however, animals are constantly aggregating new memories and updating past memories as new, relevant information is learned^22^. Moreover, most studies of associative learning have focused on cues that directly precede or occur in tandem with an outcome. However, often in nature, a predictor may not immediately precede an outcome but animals are nonetheless capable of learning to make an inference about the association (for example, conditioned taste aversion)^23^. It is unclear what conditions could promote memories to be linked across long periods (that is, hours to days), and the neural mechanisms of memory integration across such disparate time periods are poorly understood^24^. While many studies have shown that offline periods support memory consolidation, recent studies have suggested that offline periods after learning may be important for memory integration as well^25–28^.

## Retrospective memory linking across days

**Fig. 1:**
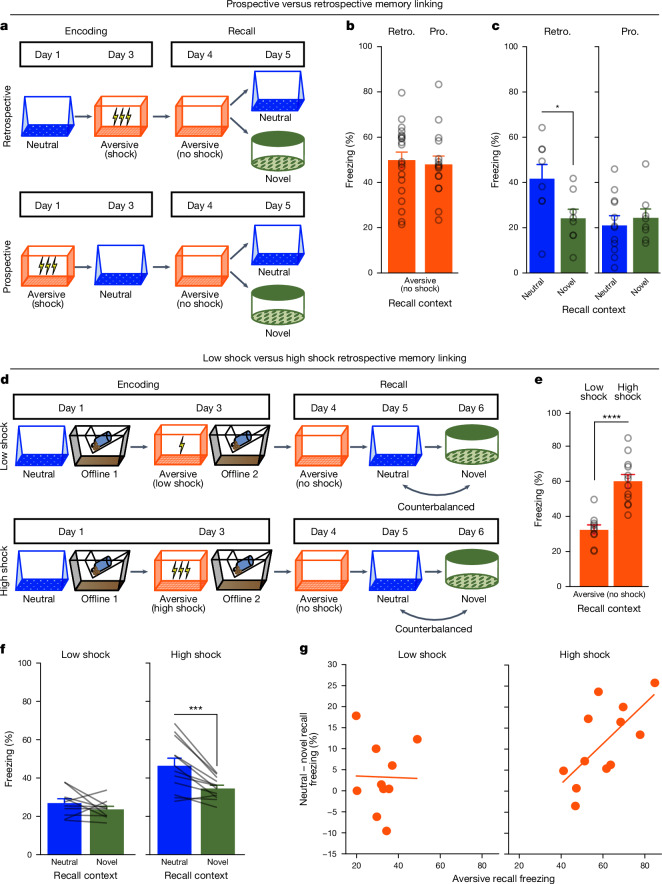
A strong aversive experience drives retrospective memory linking to a neutral context learned days ago. **a**, Schematic of the prospective versus retrospective memory-linking behaviour experiment. **b**, Freezing during aversive recall. There is no difference in aversive recall freezing between the prospective (pro.) and retrospective (retro.) conditions (*t*_34_ = 0.36, *P* = 0.72). *n* = 16 (retrospective) and *n* = 20 (prospective) mice. **c**, Freezing during neutral versus novel recall. There is a significant interaction between direction (prospective versus retrospective) and context (neutral versus novel) (*F*_1,32_ = 4.90, *P* = 0.034). *n* = 8 (retrospective neutral), *n* = 8 (retrospective novel), *n* = 12 (prospective neutral) and *n* = 8 (prospective novel) mice. Post hoc testing: retrospective (*t*_32_ = 2.586, *P* = 0.029), prospective (*t*_32_ = 0.452, *P* = 0.6546). **d**, Schematic of the low-shock versus high-shock retrospective memory-linking experiment. Calcium imaging was performed during all sessions. **e**, Freezing during aversive recall in low- versus high-shock mice. Mice froze more in the aversive context after receiving a high shock versus low shock (*t*_18,8_ = 5.877, *P* = 0.000012). *n* = 10 (low-shock) and *n* = 12 (high-shock) mice. **f**, Freezing during neutral versus novel recall in low- versus high-shock mice. There was a significant effect of context (neutral versus novel) (*F*_1,20_ = 17.32, *P* = 0.000048) and a significant interaction between context and amplitude (*F*_1,20_ = 4.99, *P* = 0.037). *n* = 10 (low shock) and *n* = 12 (high-shock) mice. High-shock mice froze more in the neutral versus novel contexts (*t*_11_ = 4.37, *P* = 0.002) and low-shock mice froze no differently (*t*_9_ = 1.23, *P* = 0.249). **g**, The correlation between aversive recall freezing and memory-linking strength. Aversive memory strength was correlated with the strength of retrospective memory linking in high-shock mice (*R*^2^ = 0.45, *P* = 0.016), but not in low-shock mice (*R*^2^ = 0.0003, *P* = 0.963). *n* = 10 (low-shock) and *n* = 12 (high-shock) mice. *P ≤ 0.05, ***P *<* 0.001, ****P < 0.0001. Error bars indicate s.e.m.

**Extended Data Fig. 1:**
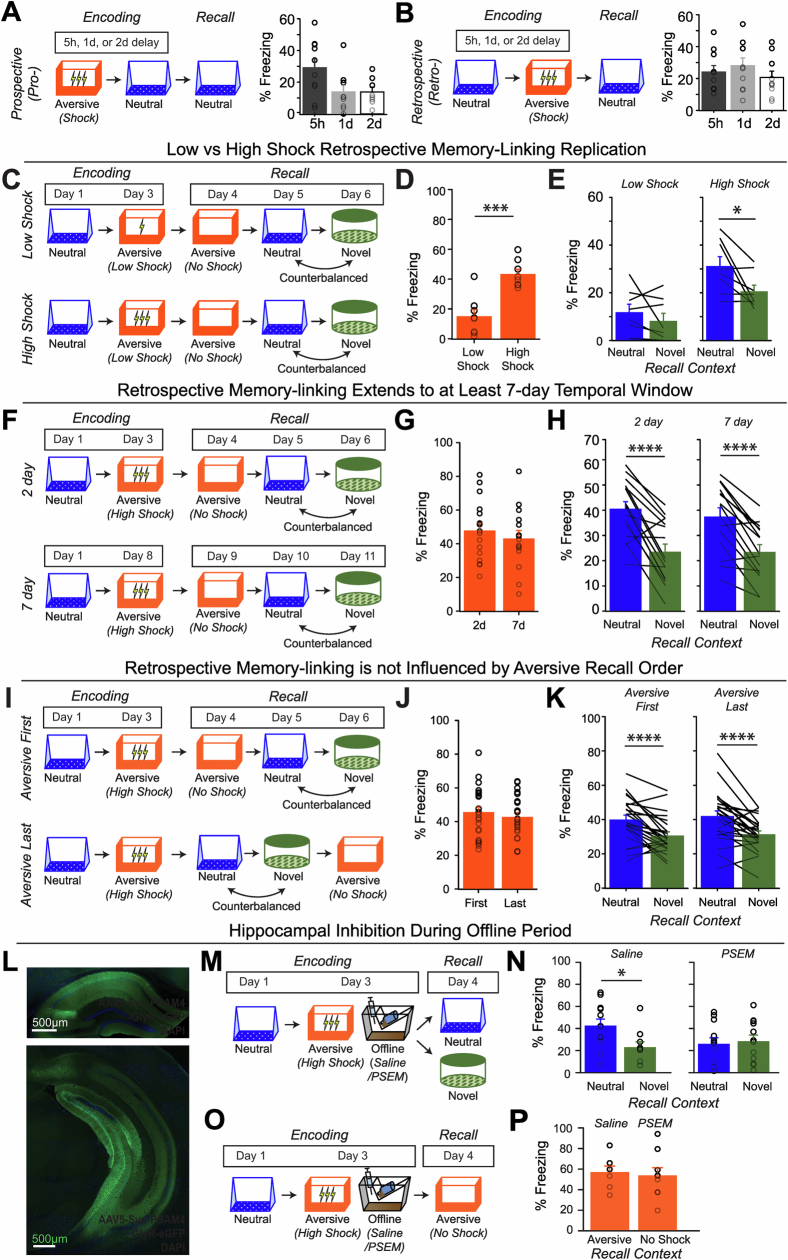
Behavioural experiment controls. A) Schematic to test the temporal window of prospective memory-linking (top). Mice underwent Aversive encoding and then either 5 h, 1d, or 2d later they underwent Neutral encoding. The following day, mice were tested in the previously experienced Neutral context. Mice froze significantly more in the Neutral context when the Neutral context occurred within 5 h of the Aversive context, compared to when it occurred one day or more after Aversive encoding (bottom). Main effect of timepoint (*F*_*2,24*_ = *3.689, p* = *0.04*) (*5* *h, n* = *10 mice; 1d, n* = *9 mice; 2d, n* = *8 mice*). Post-hoc tests revealed a trend for higher freezing in the 5 h timepoint compared to the 1d or 2d timepoints: 1d (*t*_*16.38*_ = *2.137, p* = *0.07*), 2d (*t*_*13.45*_ = *2.38, p* = *0.07*). B) Schematic to test the temporal window of retrospective memory-linking (top). Mice underwent Neutral encoding, followed by Aversive encoding in a separate context 5 h, 1d, or 2d later. The day following Aversive encoding, they were tested in the previously experienced Neutral context. Mice froze no differently in the Neutral context regardless of how long before Aversive encoding the Neutral context was experienced (bottom). No main effect of timepoint (*F*_*2,27*_ = *0.73, p* = *0.49*) (*5* *h, n* = *10 mice; 1d, n* = *10 mice; 2d, n* = *10 mice*). C) Schematic of low- vs high-shock retrospective memory-linking experiment (without calcium imaging as a replication – biological replicate). Mice underwent Neutral encoding followed by a low- or high-shock Aversive encoding two days later. In the subsequent 3 days, mice were tested in the Aversive context, and then Neutral and Novel contexts, counterbalanced. D) Mice froze more in the Aversive context in high-shock vs low-shock mice (*t*_*14*_ = *5.04, p* = *0.00018*) (*low-shock, n* = *8 mice; high-shock, n* = *8 mice*). E) High-shock mice exhibited higher freezing in Neutral vs Novel recall, while low-shock mice did not. A priori post-hoc test: *high-shock* (*t*_*7*_ = *2.65, p* = *0.033*), *low-shock* (*t*_*7*_ = *1.21, p* = *0.133*) (*low-shock, n* = *8 mice; high-shock, n* = *8 mice*). F) Schematic of temporal window retrospective memory-linking experiment to test whether memory-linking occurs at longer temporal windows. Mice underwent Neutral encoding followed by high-shock Aversive encoding two days later or seven days later. In the subsequent days, mice were tested in the Aversive context, and then Neutral and Novel contexts, counterbalanced. G) Mice froze no differently in the Aversive context in 2-day vs 7-day mice (*t*_*28.81*_ = *0.72, p* = *0.47*) (*2-day, n* = *16 mice; 7-day, n* = *15 mice*). H) Mice in both 2-day and 7-day groups showed higher freezing in Neutral vs Novel recall (*F*_*1,29*_ = *63.06, p* = *9e-9*). There was no difference in freezing in 2-day vs 7-day mice (*F*_*1,29*_ = *0.16, p* = *0.69*) and no interaction (*F*_*1,29*_ = *0.60, p* = *0.45*) (*2-day, n* = *16 mice; 7-day, n* = *15 mice*). I) Schematic to test whether the order of Aversive Recall affects retrospective memory-linking. Mice underwent Neutral encoding followed by high-shock Aversive encoding two days later. In the subsequent three days, mice were tested either in the Aversive context followed by Neutral and Novel, counterbalanced (Aversive First); or, mice were tested in Neutral and Novel, counterbalanced, followed by the Aversive context (Aversive Last). J) Mice froze no differently in the Aversive context if Aversive Recall came first or last (*t*_*46*_ = *0.72, p* = *0.48*). K) Mice in both groups (Aversive First and Aversive Last) showed higher freezing in Neutral vs Novel recall (*F*_*1,46*_ = *38.15, p* = *1.6e-7*). There was no difference in freezing in Aversive First vs Aversive Last groups (*F*_*1,46*_ = *0.19, p* = *0.66*) and no interaction (*F*_*1,46*_ = *0.14, p* = *0.71*). L) Representative histological verification of viral expression in dorsal and ventral hippocampus. Blue represents DAPI and green represents AAV5-Syn-PSAM-GFP. M) Schematic of the behavioural experiment disrupting hippocampal activity during the offline period. Mice were injected with AAV5-Syn-PSAM-GFP into dorsal and ventral hippocampus. Mice all had a Neutral experience and two days later a strong Aversive experience. Right after Aversive encoding, mice either had the hippocampus inactivated for 12hrs using the PSAM agonist, PSEM, or were given saline as a control. To do this, mice were injected four times, every three hours, to extend the manipulation across a 12-hour period. Two days later, mice were tested in the Neutral or a Novel context for freezing. N) Control (saline-treated) mice displayed retrospective memory-linking (i.e., higher freezing during Neutral vs Novel recall), while mice that received hippocampal inhibition (PSEM-treated) no longer displayed retrospective memory-linking. Significant interaction between Experimental Group (PSEM vs Sal) and Context (Neutral vs Novel) (*F*_*1,42*_ = *4.00, p* = *0.05*) (*Saline Neutral, n* = *12 mice; Saline Novel, n* = *10 mice; PSEM Neutral, n* = *12 mice; PSEM Novel, n* = *12 mice*). Post-hoc tests demonstrate higher freezing in Neutral vs Novel contexts in the Sal group (*t*_*19.84*_ = *2.57, p* = *0.03*) and no difference in freezing in Neutral vs Novel contexts in the PSEM group (*t*_*22*_ = *0.31, p* = *0.76*). O) Schematic of the behavioural experiment as above, but this time to test the effects of hippocampal inactivation on Aversive memory recall. Mice all underwent the Neutral and Aversive experiences as before, as well as PSEM or saline injections following Aversive encoding (as in Extended Data Fig. 1m); however, two days following Aversive encoding, mice were tested in the Aversive context to test for an intact aversive memory. P) Mice froze no differently in the Aversive context whether they had received hippocampal inhibition or not (*t*_*13.9*_ = *0.32, p* = *0.748*) (*Saline, n* = *7 mice; PSEM, n* = *9 mice*).

To investigate how memories are integrated across days, we first designed a behavioural experiment to test whether mice would spread fear from an aversive memory to a neutral memory formed 2 days before (retrospective memory linking) or 2 days after (prospective memory linking) (Fig. 1a). In the retrospective group, mice first experienced a neutral context followed by an aversive context paired with a foot shock 2 days later. In the prospective group, the mice experienced an aversive context followed by a neutral context 2 days later. Both groups were then tested in the aversive context to test for recall of the aversive memory. They were then tested in either the previously experienced neutral context for memory linking or an unfamiliar novel context to test for non-specific fear generalization. Memory linking was defined as a selective increase in fear in the neutral context compared with in the novel context, both contexts in which mice had never been shocked. Notably, this definition distinguishes memory linking to a specific context from non-specific generalization of fear. There was no difference in freezing in the aversive context between groups (Fig. 1b), suggesting that the perceived negative valence of the aversive context was not different between groups. In the retrospective group, mice froze more in the neutral context compared with in the novel context, suggesting that fear spread retrospectively from the aversive context to the neutral context experienced 2 days before. However, in the prospective group, there was no difference in freezing between the neutral and novel contexts, suggesting that memory linking between the aversive and neutral contexts did not occur prospectively across days (Fig. 1c). Consistent with previous studies demonstrating memory linking when memories are encoded within a day^29,30^, we also observed memory linking with neutral and aversive contexts when separated by 5 h (Extended Data Fig. 1a,b).

**Extended Data Fig. 2:**
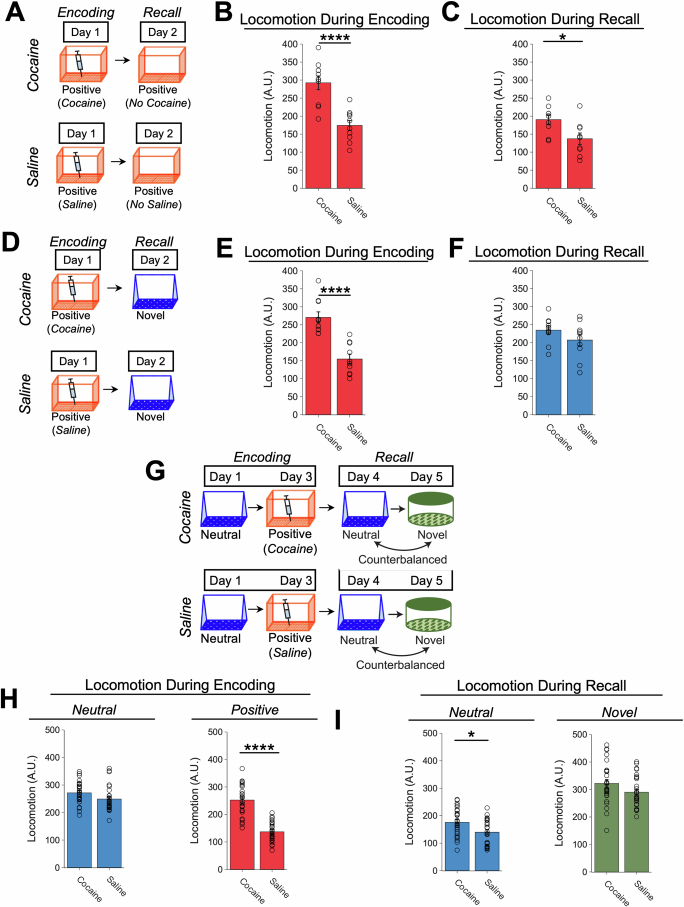
Retrospective memory-linking with an appetitive contextual memory. A) Schematic of behavioural experiment to test whether cocaine-context pairing leads to a measurable conditioned response in the conditioned context. Mice were administered cocaine or saline immediately prior to exposure to a novel context. The following day, they were returned to the conditioned context off-drug for recall. B) Mice that received cocaine locomoted significantly more than saline controls during encoding (*t*_*18*_ = *5.07, p* = *0.00008*) (*Cocaine, n* = *9 mice; Saline, n* = *9 mice*). C) Mice that received cocaine locomoted significantly more than saline controls during recall in the conditioned context the day following encoding (*t*_*16*_ = *2.92, p* = *0.010*) (*Cocaine, n* = *9 mice; Saline, n* = *9 mice*). D) Schematic of behavioural experiment to test whether the conditioned response observed in Extended Data Fig. 2a–c is context-specific. Mice were administered cocaine or saline immediately prior to exposure to a novel context. The following day, they were placed in a novel context. E) Mice that received cocaine locomoted significantly more than saline controls during encoding (*t*_*18*_ = *5.64, p* = *0.000024*) (*Cocaine, n* = *10 mice; Saline, n* = *10 mice*). F) Mice that received cocaine locomoted no differently than saine controls during recall of a novel context (*t*_*18*_ = *1.35, p* = *0.20*) (*Cocaine, n* = *10 mice; Saline, n* = *10 mice*). G) Schematic of behavioural experiment to test for retrospective memory-linking with cocaine. Mice were exposed to a neutral context, and two days later they were administered either cocaine or saline immediately prior to being placed in a separate context. In the subsequent days, mice were tested in Neutral and Novel contexts, counterbalanced, and then in the cocaine-paired context last. H) Left: Mice locomoted no differently in the Neutral encoding context (*t*_*54*_ = *1.96, p* = *0.056*). Right: Mice that received cocaine locomoted more than mice that received saline during Cocaine encoding (*t*_*54*_ = *9.36, p* = *6.72e-13*) (*Cocaine, n* = *28 mice; Saline, n* = *28 mice*). I) Left: There is a strong trend that mice that received cocaine locomoted more during Neutral recall than mice that received saline (*t*_*54*_ = *2.85, p* = *0.01*). Right: Mice that received cocaine or saline locomoted no differently in Novel recall (*t*_*54*_ = *1.83, p* = *0.07*) (*Cocaine, n* = *28 mice; Saline, n* = *28 mice*).

We next examined what conditions drove memories to be linked retrospectively across days. It has previously been suggested that the emotional salience of an experience enhances its storage into memory^31^, as well as its likelihood of altering past neutral memories in humans^32^. We therefore hypothesized that the more aversive the experience, the more likely fear would be retrospectively linked to a previous neutral memory. To test this, we manipulated the shock intensity during aversive encoding to test whether a stronger shock would drive greater retrospective memory linking (Fig. 1d and Extended Data Fig. 1). Mice were exposed to a neutral context followed by an aversive context paired with a low-amplitude (0.25 mA) or high-amplitude (1.5 mA) shock 2 days later (low-shock group and high-shock group). The mice were then tested in the aversive, neutral and novel context on the subsequent 3 days. As expected, the high-shock group froze more than the low-shock group during recall in the aversive context (Fig. 1e). Next, we found that the high-shock group exhibited an increase in freezing in the previously experienced neutral context relative to the novel context, but the low-shock group did not (Fig. 1f and Extended Data Fig. 1c–e). If the perceived aversiveness of an experience affects the likelihood of retrospective memory linking, we hypothesized that the levels of freezing during aversive memory recall would positively correlate with memory linking—defined as the difference between freezing in the neutral context and in the novel context. Indeed, in the high-shock mice, freezing during aversive context recall was positively correlated with the degree of memory linking (Fig. 1g). These data suggest that a strong aversive experience can retrospectively link with neutral memories formed days before (up to 7 days, Extended Data Fig. 1f–h). Retrospective memory linking was not influenced by the order in which recall sessions occurred (Extended Data Fig. 1i–k). Moreover, we also found evidence that a highly salient, appetitive experience (that is, cocaine exposure) also drove retrospective memory linking to a neutral context memory formed 2 days before, suggesting that retrospective memory linking may be a broad mechanism for updating salient memories encoded across days (Extended Data Fig. 2).

We next investigated how the brain links recent aversive memories with past neutral memories formed days before. It has been well established in rodents and humans that memories are reactivated during restful periods after learning (that is, offline periods) to promote the storage of recently learned information^13–15^. Moreover, recent work in humans has shown that offline periods can drive the integration of discrete memories as well^25,33,34^. We therefore hypothesized that after an aversive experience (high-shock group), the offline period may function not only to support the consolidation of the aversive memory, but also to link the recent aversive memory with the previous neutral memory, therefore increasing freezing during recall of the neutral context. A major site of memory formation in the brain is the hippocampus, where rapid plasticity after an experience promotes the formation of a memory for that experience and reflects memory expression thereafter^6,13,15,35^. Thus, we used a chemogenetic system to disrupt endogenous hippocampal activity during the offline period after aversive encoding. We found that this prevented retrospective memory linking (that is, selective freezing in the neutral context compared with in the novel context) while leaving the aversive memory intact (Extended Data Fig. 1l–p), suggesting that the hippocampus has a critical role in retrospective memory linking.

## Offline reactivation of a past neutral ensemble

Previous research has suggested that memory reactivation during offline periods after learning could promote not only the consolidation of recently formed memories, but also support the integration of memories^25,27,28,33,34,36^. Thus, we expected that the hippocampal ensemble that was active during aversive encoding would be reactivated during the offline period to drive consolidation of the recently learned aversive memory. Moreover, we hypothesized that if the aversive experience was strong enough, the ensemble active during the neutral experience (from 2 days before) would be reactivated as well, driving integration of the neutral and aversive memories.

**Extended Data Fig. 3:**
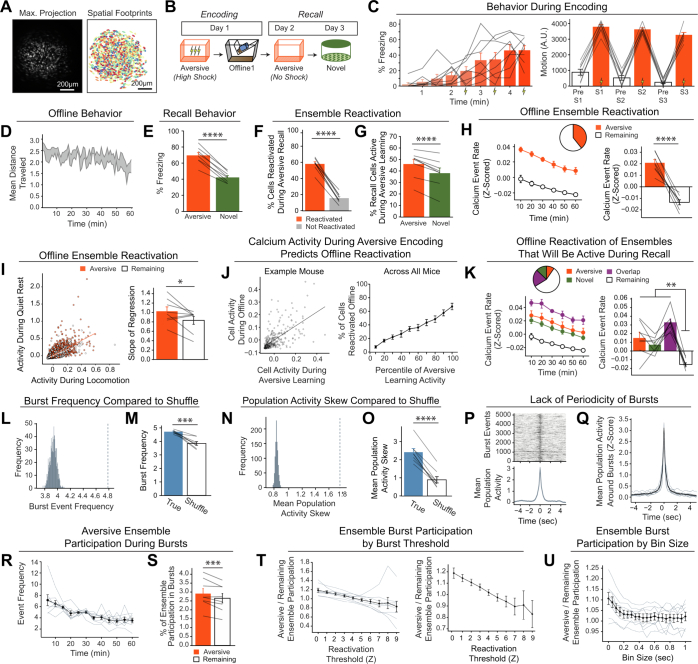
Neurons active during Aversive encoding are selectively reactivated offline and during Aversive recall. A) Representative maximum intensity projection of the field-of-view of one example session (left). Spatial footprints of all recorded cells during the session, randomly colour-coded (right). B) Schematic of a single aversive experience. Mice had an Aversive experience followed by a 1 hr offline session in the homecage. The next day, mice were tested in the Aversive context, followed by a test in a Novel context one day later. Calcium imaging in hippocampal CA1 was performed during all sessions. C) Mice acquired within-session freezing during Aversive encoding (left); main effect of time (*F*_*8,56*_ = *12.59, p* = *3.87e-10, n* = *8 mice*). And mice responded robustly to all three foot shocks, though their locomotion generally decreased across shocks, driven by increased freezing (right); main effect of shock number (*F*_*2,14*_ = *7.45, p* = *0.0154, n* = *8 mice*) and main effect of PreShock vs Shock (*F*_*1,7*_ = *581, p* = *5.38e-8, n* = *8 mice*), and no interaction. D) Mice displayed a modest decrease in locomotion across the 1 hr offline period (arbitrary units) (*R*^*2*^ = *0.064, p* = *1.9e-8, n* = *8 mice*). E) Mice froze significantly more in the Aversive context than in a Novel context during recall (*t*_*7*_ = *165, p* = *4e-6, n* = *8 mice*). F) Cells that were active during Aversive encoding and reactivated offline were significantly more likely to be reactivated during Aversive recall than cells active during Aversive encoding and not reactivated offline (*t*_*7*_ = *19.41, p* = *2e-7, n* = *8 mice*). G) A larger fraction of cells active during Aversive recall than during Novel recall were previously active during Aversive encoding (*t*_*7*_ = *6.897, p* = *0.0002, n* = *8 mice*). H) During the offline period, ~40% of the population was made up of cells previously active during Aversive encoding (top). This Aversive ensemble was much more highly active than the rest of the population during the offline period (bottom; A.U.) (*t*_*7*_ = *8.538, p* = *0.00006, n* = *8 mice*). I) Each cell’s activity was compared during locomotion vs during quiet rest (left; A.U.). A regression line was fit to the cells in the Aversive ensemble and in the Remaining ensemble separately, for each mouse. The Remaining ensemble showed greater activity during locomotion than during quiet rest (i.e., a less positive slope). The Aversive ensemble showed relatively greater activity during quiet rest than locomotion (i.e., a more positive slope) across mice (right) (*t*_*7*_ = *5.76, p* = *0.047, n* = *8 mice*). J) Cells that had high levels of activity (A.U.) during Aversive encoding continued to have high levels of activity during the offline period (example mouse; left). There was a linear relationship between how active a cell was during Aversive encoding and how likely it was to be reactivated during the offline period (all mice; right) (*R*^*2*^ = *0.726, p* = *1.25e-23, n* = *8 mice*). K) During the offline period, cells that would go on to become active during recall were more highly active than the Remaining ensemble during the offline period. The top represents the proportion of each ensemble (legend to its right). The cells that would become active during both Aversive and Novel recall were most highly active (A.U.). There was no difference in activity in the cells that would go on to be active in Aversive or Novel. Main effect of Ensemble (*F*_*3,21*_ = *27.81, p* = *1.65e-7, n* = *8 mice*). Post-hoc tests: for Aversive vs Novel (*t*_*7*_ = *1.33, p* = *0.22*), for Remaining vs Aversive ∩ Novel (*t*_*7*_ = *11.95, p* = *0.000007*), for Remaining vs Aversive (*t*_*7*_ = *3.97, p* = *0.005*), for Remaining vs Novel (*t*_*7*_ = *7.47, p* = *0.0001*). L) Neuron activities were circularly shuffled 1000 times relative to one another and the mean population activity was re-computed each time. This shuffling method preserved the autocorrelations for each neuron while disrupting the co-firing relationships between neurons. The burst frequency was computed for each of these shuffles to produce a shuffled burst frequency distribution (grey histogram), to which the true burst frequency was compared (blue dotted line). This is an example mouse. M) The mean burst frequency for the shuffled distribution was computed and compared to the true burst frequency for each mouse. True burst frequencies were greater than shuffled burst frequencies in every mouse (*t*_*7*_ = *6.159, p* = *0.000463, n* = *8 mice*), suggesting that during the offline period, hippocampal CA1 neurons fire in a more coordinated manner than would be expected from shuffled neuronal activities. N) As in Extended Data Fig. 3l, neuron activities were shuffled, and mean population was re-computed each time. From this population activity trace, the skew of the distribution was computed. If there were distinct periods where many neurons simultaneously fired, we hypothesized that the true distribution of mean population activity would be more skewed with a strong right tail demonstrating large and brief deflections, compared to shuffled neuronal activities. We computed the skew of each shuffled mean population activity, to produce a distribution (grey histogram), to which the true mean population’s skew was compared (blue dotted line). This is an example mouse. O) The mean skew for the shuffled distribution was computed and compared to the true skew of the mean population activity for each mouse. The true skew was greater than the shuffled skew in every mouse (*t*_*7*_ = *13.36, p* = *0.000003, n* = *8 mice*), supporting the idea that the mean population activity undergoes brief burst-like activations requiring the coordinated activity of groups of neurons. P) Matrix of burst events for an example mouse, stacked along the y-axis and centred on time t = 0 (top), and the average mean population activity around each burst event (bottom). Q) As in Extended Data Fig. 3p but averaged across all mice. Each thin line represents one mouse, and the thick black line represents the mean across mice with the grey ribbon around it representing the standard error (*n* = *8 mice*). There is no periodicity to when these burst events occur. R) The burst event frequency decreased across the hour (*F*_*11,77*_ = *6.91, p* = *5.66e-8, n* = *8 mice*). S) A larger fraction of the Aversive ensemble vs the Remaining ensemble participated in each burst event (left) (*t*_*7*_ = *3.68, p* = *0.0079, n* = *8 mice*). T) Ensemble burst participation as a function of burst threshold. The burst threshold was parametrically varied, and the ratio of Aversive-to-Remaining burst participation was computed at each burst threshold. Aversive-to-Remaining burst ratio is negatively related to burst threshold (*R*^*2*^ = *0.28, p* = *3.4e-7*) (*n* = *8 mice*). On the left graph, the black line represents the mean across mice with SEM represented in the error bars, and each individual mouse is represented by the grey lines. On the right is the same data as on the left graph, but without the individual mice. U) Ensemble burst participation as a function of bin size. The Aversive-to-Remaining burst participation ratio was computed at varying bin sizes. At larger bin sizes, the selective increase in Aversive burst participation is no longer present (*n* = *8 mice*).

We first validated that we could detect ensemble reactivation after a salient experience using calcium imaging. To do this, we conducted a contextual fear conditioning experiment, recording hippocampal CA1 calcium dynamics using the open-source UCLA Miniscope^29^ (Extended Data Fig. 3). We recorded during aversive encoding, the first hour offline after aversive encoding, and during recall of the aversive context and exposure to a novel context. Consistent with previous literature, we found that the ensemble of cells active during aversive encoding was reactivated offline and preferentially reactivated during aversive memory recall, suggesting a stable neural memory ensemble (Extended Data Fig. 3a–k).

**Fig. 2:**
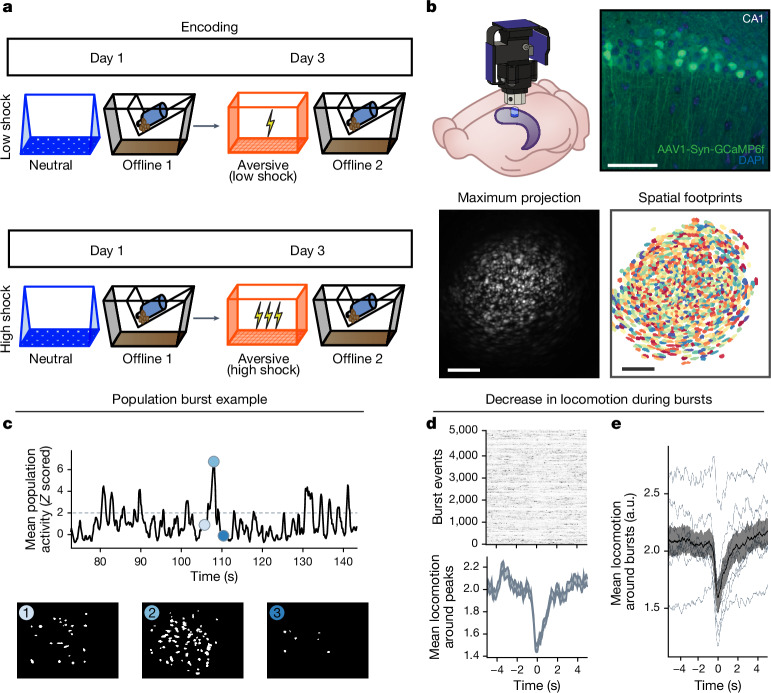
Hippocampal ensembles exhibit population bursts of calcium events during offline periods. **a**, Behavioural schematic of the retrospective memory-linking experimental design. The same as in Fig. 1d, but focusing here on the offline periods. **b**, Schematic of the lens and Miniscope placement onto the dorsal hippocampus (top left). Top right, representative histological analysis of GCaMP6f expression in the hippocampal CA1, imaged using confocal microscopy. Green, AAV1-Syn-GCaMP6f expression; blue, cellular DAPI stain. Bottom left, maximum-intensity projection of an example mouse across one recording session. Bottom right, spatial footprints of all recorded cells during the session on the left randomly colour coded. This experiment was repeated across two cohorts. Scale bars, 50 μm (top) and 200 μm (bottom). **c**, Example of a burst event. The top trace represents the *z*-scored mean population activity within one of the offline recordings. Three timepoints were chosen (overlaid in circles), the middle representing the peak of a burst event and the timepoints to its left and right representing *t* − 2 s and *t* + 2 s from the peak, respectively. The bottom three matrices represent binarized spatial footprints depicting the spatial footprints of the cells sufficiently active to participate in a burst (*z* > 2). The matrices represent the timepoints of the three datapoints above it, ordered by time. **d**, Locomotion of an example mouse during each burst event stacked along the *y* axis (top), and the mean locomotion around burst events (bottom). Mice showed a robust and brief slowing down around 1 s before each burst event, before increasing locomotion back up around 2 s later. **e**, Mouse locomotion as in **d**, but averaged across all of the mice. Each thin line represents one mouse, and the thick black line represents the mean across mice, with the grey ribbon around it representing the s.e.m. *n* = 8 mice. This demonstrates a robust and reliable decrease in locomotion around the onset of burst events. From the experiment in Extended Data Fig. 3. Error bands indicate s.e.m.

**Extended Data Fig. 4:**
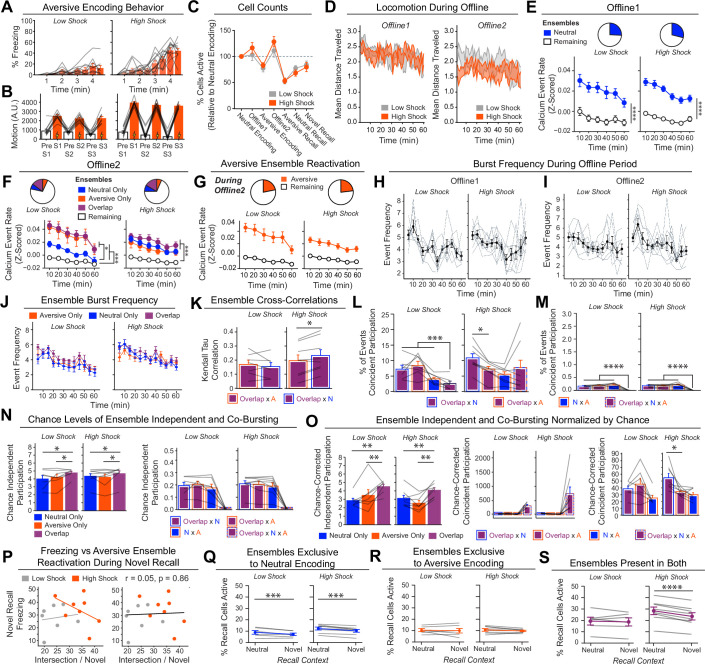
Low- vs High-shock calcium imaging supplementary analyses. A) Mice acquired within-session freezing during Aversive encoding. Mice that received high shocks (1.5 mA) displayed more freezing than mice that received low shocks (0.25 mA) (*low-shock, n* = *10 mice; high-shock, n* = *12 mice*). B) Mice responded robustly to each foot shock. High-shock mice responded more strongly to each shock than low mice did (*low-shock, n* = *10 mice; high-shock, n* = *12 mice*). C) Relative to the first calcium imaging recording, mice showed comparable fractions of observed cells across the remaining sessions (*low-shock, n* = *8 mice; high-shock, n* = *10 mice*). D) Locomotion across the 1 hr offline period after Neutral encoding (Offline 1) and after Aversive encoding (Offline 2) in low- and high-shock mice (in arbitrary units). Mice showed decreased locomotion across the offline period on both days. Low Shock mice did not locomote differently from high-shock mice during either offline period (*low-shock, n* = *10 mice; high-shock, n* = *12 mice*). E) During Offline 1 after Neutral encoding, cells that were active during Neutral encoding (Neutral ensemble) made up ~25-30% of the offline cell population (pie charts) (*X*^*2*^ = *0.122, df* = *1, p* = *0.73*). The Neutral ensemble was more highly active than the Remaining ensemble during the offline period (line graphs; A.U.). There was a main effect of Ensemble (*F*_*1,159*_ = *59.19, p* = *1.4e-12*), no effect of Amplitude (*F*_*1,13*_ = *0.039, p* = *0.85*), and an effect of Time (*F*_*1,159*_ = *4.33, p* = *0.039*), and all interactions *p* > *0.05* (*low-shock, n* = *7 mice; high-shock, n* = *8 mice; 659 Offline 1 cells recorded per mouse on average*). F) During Offline 2 after Aversive encoding, similar proportions of previously active cells were reactivated across low- and high-shock groups (pie charts) (*X*^*2*^ = *0.326, df* = *3, p* = *0.955*). However, ensembles were differentially reactivated based upon the amplitude of the Aversive experience (*Ensemble x Amplitude: F*_*3,331*_ = *5.36, p* = *0.0013*) (line graphs; A.U.). In low-shock mice, the Neutral, Aversive, and Overlap ensembles were more highly active than the Remaining ensemble (*contrast, t*_*18*_ = *4.22, p* = *0.0005*). Additionally, these ensembles were differentially active relative to one another (*F*_*2,12*_ = *4.03, p* = *0.046*). This was driven by the Neutral ensemble being less active. The Neutral ensemble was less active than the Aversive and Overlap ensembles (*t*_*12*_ = *2.83, p* = *0.03*) while the Aversive ensemble was no differently active than the Overlap ensemble (*t*_*12*_ = *0.19, p* = *0.85*). In high-shock mice, the Neutral, Aversive, and Overlap ensembles were all more highly active than the Remaining ensemble (*t*_*21*_ = *4.36, p* = *0.0003*), but these three ensembles were no differently active from each other (*F*_*2,14*_ = *1.52, p* = *0.25*). In high-shock mice compared to low-shock mice, the Overlap and Aversive ensembles were less active than in high-shock mice (Overlap ensemble: *t*_*75.43*_ = 2.44, p = 0.03; Aversive ensemble: *t*_*65.83*_ = 3.59, p = 0.003). (*low-shock, n* = *7 mice; high-shock, n* = *8 mice; 705 Offline 2 cells recorded per mouse on average*). G) Aversive ensemble reactivation compared to Remaining ensemble during Offline 2. Ensemble reactivation here is measured as it is during Offline 1 following Neutral encoding (Extended Data Fig. 4e). There is a significant effect of ensemble (Aversive vs Remaining) (*F*_*1,159*_ = *90.14, p* = *0.00*). There is a significant effect of time (*F*_*1,159*_ = *4.05, p* = *0.046*). There is no significant effect of Amplitude (low vs high-shock) (*F*_*1,13*_ = *0.045, p* = *0.84*) (*low-shock, n* = *7 mice; high-shock, n* = *8 mice*). H) During Offline 1, burst event frequency gradually decreased across the hour (*F*_*11,143*_ = *4.43, p* = *1.0e-5*). No difference across shock amplitudes (*F*_*11,13*_ = *0.31, p* = *0.587*) (*low-shock, n* = *7 mice; high-shock, n* = *8 mice*). Significant interaction between Time and Amplitude (*F*_*11,143*_ = *1.87, p* = *0.047*). Follow-up repeated measures ANOVAs showed that both low- and high-shock groups showed a significant decrease in event rate across time (*low-shock: F*_*11,66*_ = *4.13, p* = *0.0001; high-shock:* (*F*_*11,77*_ = *2.43, p* = *0.01*). I) During Offline 2, burst event frequency decreased across time (*F*_*11,143*_ = *6.69, p* = *0.000054*). No difference across shock amplitudes (*F*_*1,13*_ = *0.0056, p* = *0.94*) (*low-shock, n* = *7 mice; high-shock, n* = *8 mice*). J) During Offline 2, bursts as defined by each ensemble (rather than by whole population) decreased across the hour, with comparable frequencies across ensembles and amplitudes (*low-shock, n* = *7 mice; high-shock, n* = *8 mice*). K) Time-lagged cross correlations between the Overlap ensemble and the Neutral and Aversive ensembles during the offline period. Each of the three ensembles (Overlap, Neutral, and Aversive) were binned into 120 sec bins. Each time bin of Overlap ensemble activity was cross-correlated with the corresponding time bin of Neutral ensemble and Aversive ensemble activity. Cross-correlations were computed with a maximum time lag of 5 frames (or, ~160 ms). For each mouse, the correlations were averaged across all time bins to get an average cross-correlation between the Overlap ensemble and Neutral ensemble (i.e., Overlap x Neutral) and the Overlap ensemble by Aversive ensemble (i.e., Overlap x Aversive). There was a significant interaction between Ensemble Combination and low- vs high-shock group (*F*_*1,13*_ = *6.70, p* = *0.02*) (*low-shock, n* = *7 mice; high-shock, n* = *8 mice*). Post-hoc tests revealed that in high-shock mice, Overlap x Neutral correlations were higher than Overlap x Aversive correlations (*t*_*7*_ = *3.97, p* = *0.01*) whereas they were no different in low-shock mice (*t*_*6*_ = *0.83, p* = *0.44*). L) As in Fig. 4d, the whole population was used to define bursts and the z-scored mean population activities were used to define participation of each ensemble. Co-participation was defined as a whole population burst during which multiple ensembles participated simultaneously. There were four possible combinations (from left to right: Overlap x Neutral, Overlap x Aversive, Neutral x Aversive, Overlap x Neutral x Aversive). During burst periods, there was a significant interaction between Ensemble Combination and low- vs high-shock (*p* = *0.01*), suggesting that the patterns of co-bursting varied in low- vs high-shock mice. Post-hoc tests revealed that in low-shock mice, co-participation between all 3 ensembles was less likely to occur than the other combinations (*t*_*18*_ = *4.73, p* = *0.0003*), while in high-shock mice, co-participation between all 3 ensembles occurred no differently than the other combinations (*t*_*21*_ = *0.358, p* = *0.72*). Additionally, in the high-shock group, the Overlap ensemble preferentially co-participated with the Neutral ensemble compared to with the Aversive ensemble (*t*_*21*_ = *2.373, p* = *0.05*), whereas in the low-shock group, the Overlap ensemble participated no differently with the Neutral and Aversive ensembles (*t*_*18*_ = *1.196, p* = *0.25*) (*low-shock, n* = *7 mice; high-shock, n* = *8 mice*). M) During non-burst periods, co-participation between all 3 ensembles was less likely than the other combinations (*t*_*39*_ = *10.92, p* = *1.98e-13*); however, there was no effect of low- vs high-shock (*F*_*1,13*_ = *0.038, p* = *0.847*) and no interaction (*F*_*3,39*_ = *0.198, p* = *0.897*) (*low-shock, n* = *7 mice; high-shock, n* = *8 mice*). N) Chance levels of ensemble independent and co-bursting. Left: The chance levels of independent bursting of the Overlap ensemble was higher than Neutral or Aversive independent bursting (F_2,26_ = 6.61, p = 0.005). There was no difference between low- vs high-shock groups (*F*_*1,13*_ = *0.030, p* = *0.87*) and no interaction (*F*_*2,26*_ = *0.96, p* = *0.40*). Right: Chance levels of triple co-bursting was much lower than of any combination of two ensembles (*F*_*3,39*_ = *98.3, p* = *3e-18*). There was no difference between low- vs high-shock groups (*F*_*1,13*_ = *0.07, p* = *0.79*) and no interaction (*F*_*3,39*_ = *0.20, p* = *0.90*) (*low-shock, n* = *7 mice; high-shock, n* = *8 mice*). O) Ensemble independent and co-bursting normalized by chance. Left: Overlap independent bursting was higher than Neutral or Aversive independent bursting (*F*_*2,26*_ = *13.82, p* = *0.00008*). There was no difference between low- vs high-shock groups (*F1,13* = *0.69, p* = *0.42*) and no interaction (*F*_*2,26*_ = *2.2, p* = *0.13*). Right: Overlap x Neutral co-bursting was more likely to occur than Overlap x Aversive co-bursting in high-shock mice (*t*_*7*_ = *3.06, p* = *0.043*) but not in low-shock mice (*t*_*6*_ = *1.25, p* = *0.26*) (*low-shock, n* = *7 mice; high-shock, n* = *8 mice*). P) Correlation between Aversive/Novel ensemble overlap with Novel recall freezing. Left: separate regression lines for low- vs high-shock mice. Right: one regression line for all mice. There was no correlation between Aversive/Novel ensemble overlap and Novel recall freezing (*R*^*2*^ = *0.005, p* = *0.86*) (*low-shock, n* = *7 mice; high-shock, n* = *8 mice*). Q) Cells active only during the Neutral experience and not the Aversive experience were more likely to be reactivated when mice were placed back in the Neutral context, compared to when they were placed in a Novel context (*F*_*1,12*_ = *24.44, p* = *0.0003*). There was no effect of shock amplitude (*F*_*1,12*_ = *3.08, p* = *0.10*) (*low-shock, n* = *6 mice; high-shock, n* = *8 mice*). R) Cells active during the Aversive experience and not the Neutral experience were no differently reactivated in Neutral vs Novel contexts. (*Amplitude: F*_*1,12*_ = *0.029, p* = *0.869; Context: F*_*1,12*_ = *1.39, p* = *0.261; Amplitude x Context: F*_*1,12*_ = *0.14, p* = *0.71*) (*low-shock, n* = *6 mice; high-shock, n* = *8 mice*). S) Cells active during both the initial Neutral and Aversive experiences were subsequently more likely to be reactivated in the Neutral context compared to Novel context in high-shock mice (*t*_*7*_ = *8.53, p* = *0.00012*), but not low-shock mice (*t*_*5*_ = *0.55, p* = *0.61; Context x Amplitude: F*_*1,12*_ = *10.33, p* = *0.007)* (*low-shock, n* = *6 mice; high-shock, n* = *8 mice*).

Next, to investigate whether a strong aversive experience was driving offline reactivation of ensembles representing both the aversive and neutral memories, we performed calcium imaging recordings in the CA1 while mice underwent the retrospective memory-linking paradigm (Fig. 1d). Here we focused on the offline periods after the initial neutral experience (offline 1) and subsequent aversive experience (offline 2) in both the low- and high-shock groups (Fig. 2a,b and Extended Data Fig. 4; the same experiment as in Fig. 1d). Consistent with the literature^13,18^ and with our previous experiment (Extended Data Fig. 3), after the initial neutral encoding (offline 1), the cells that were active during that neutral encoding (neutral ensemble) were more active than cells that were not active during neutral encoding (remaining ensemble) in both the low- and high-shock groups (Extended Data Fig. 4e). There was no difference in the number of neutral ensemble cells that were active during offline 1 between the low- and high-shock groups (Extended Data Fig. 4e). To measure ensemble reactivation during the offline period after aversive encoding (offline 2), we sorted cells that were active during the offline period into four ensembles on the basis of when those cells were previously active: a ‘neutral’ ensemble comprising cells that were active during the initial neutral encoding and not during aversive encoding; an ‘aversive’ ensemble comprising cells that were active during aversive encoding but not during neutral encoding; an ‘overlap’ ensemble comprising cells that were active during both neutral and aversive encoding; and a ‘remaining’ ensemble comprising cells that were not observed to be active before the offline period (Extended Data Fig. 4f). There was no difference in the proportion of cells that made up each ensemble across the low- and high-shock groups (Extended Data Fig. 4f). In the low-shock group, we found that the aversive ensemble, the neutral ensemble and the overlap ensemble had higher calcium activity than the remaining ensemble. However, the neutral ensemble was less active than the aversive and overlap ensembles (Extended Data Fig. 4f (left)). These results are consistent with previous studies demonstrating that neuronal ensembles from recent memories are reactivated offline^13–15^. By contrast, in the high-shock group, the neutral ensemble was no differently active than the aversive and overlap ensembles (Extended Data Fig. 4f (right)), indicating that the high shock increased reactivation of the neutral ensemble. Notably, we also found that the activity of the aversive and overlap ensembles was lower in high-shock mice compared with in low-shock mice. This is consistent with the idea that homeostatic mechanisms may have a role in regulating overall activity in hippocampus, consistent with past reports across brain areas^37,38^, such that if the neutral ensemble becomes more highly active, the other highly active cells must become less active.

## Neutral ensemble recruited into population bursts

Since the neutral ensemble was more highly reactivated after a high shock, we next investigated whether the neutral, aversive and overlap ensembles might be firing together on a finer temporal scale. Hippocampal activity is known to exhibit organized bursts, often accompanied by sharp-wave ripples in the local field potential, during which cells that are active during learning are preferentially reactivated^13–15^. These events have been found to support memory consolidation^13,18–21^. Although calcium dynamics are of a coarser timescale than burst events recorded electrophysiologically, we observed that during the offline recordings, hippocampal calcium events periodically exhibited brief bursts of activity during which numerous cells were co-active (Fig. 2c), consistent with previous reports^39,40^. Notably, these burst events coincided with the mouse briefly slowing down about 1 s before the burst event, and about 1 s after, resuming its locomotion (Fig. 2d,e). This suggests that these burst events occurred during brief periods of quiescence^13^. We found that these bursts were unlikely to occur from shuffled neuronal activities, suggesting that these were organized events (that is, when groups of hippocampal neurons were synchronously active; Extended Data Fig. 3l–o). We isolated these brief burst periods to examine whether ensembles that were previously active during encoding were preferentially participating in these brief burst events. We first measured burst events during the offline period after an aversive experience and found that a larger proportion of aversive ensemble cells participated in these burst events than the remaining ensemble cells (Extended Data Fig. 3s).

**Fig. 3:**
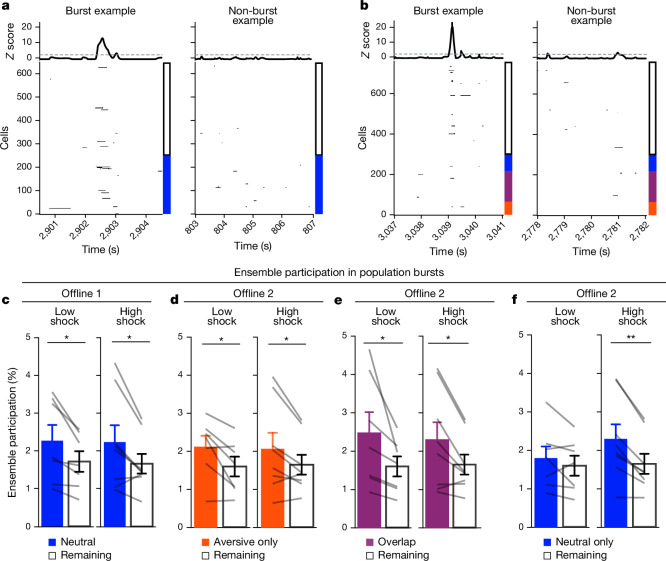
A strong aversive experience recruits the past neutral ensemble into offline population bursts. **a**, Example offline 1 burst event (left). Each row represents the activity of a neuron, colour coded by ensemble (blue, neutral; white, remaining). The top black trace represents the *z*-scored mean population activity. Right, example non-burst event. **b**, The same as in **a** but for offline 2 (red, aversive; purple, overlap; blue, neutral; white, remaining). **c**, During offline 1 in the low- and high-shock groups, a greater percentage of the neutral ensemble participated in bursts than the remaining ensemble (ensemble: *F*_1,13_ = 16.33, *P* = 0.001; amplitude: *F*_1,13_ = 0.009, *P* = 0.925; ensemble × amplitude: *F*_1,13_ = 0.0058, *P* = 0.940). *n* = 7 (low-shock) and *n* = 8 (high-shock) mice. **d**, During offline 2 in the low- and high-shock groups, a greater percentage of the aversive ensemble participated in bursts than the remaining ensemble (ensemble: *F*_1,13_ = 13.57, *P* = 0.0028; amplitude: *F*_1,13_ = 0.000078, *P* = 0.99; ensemble × amplitude: *F*_1,13_ = 0.16, *P* = 0.69). *n* = 7 (low-shock) and *n* = 8 (high-shock) mice. **e**, During offline 2 in the low- and high-shock groups, a greater percentage of the overlap ensemble participated in bursts than the remaining ensemble (ensemble: *F*_1,13_ = 13.95, *P* = 0.0025; amplitude: *F*_1,13_ = 0.014, *P* = 0.91; ensemble × amplitude: *F*_1,13_ = 0.31, *P* = 0.58). *n* = 7 (low-shock) and *n* = 8 (high-shock) mice. **f**, During offline 2, neutral and remaining ensembles differentially participated in bursts in the high- and low-shock groups (ensemble × amplitude: *F*_1,13_ = 5.186, *P* = 0.040). High-shock mice showed higher neutral ensemble participation relative to the remaining ensemble (*t*_7_ = 4.88, *P* = 0.0036), low-shock mice showed no difference in ensemble participation (*t*_6_ = 1.33, *P* = 0.23). *n* = 7 (low-shock) and *n* = 8 (high-shock) mice. **P < 0.01. Error bars indicate s.e.m.

We then examined whether a strong aversive experience drove the neutral ensemble to also participate in these bursts after aversive encoding (Fig. 3). Frequencies of burst events were comparable across groups and decreased across the hour during the offline periods after both neutral (offline 1) and aversive (offline 2) encoding (Extended Data Fig. 4h,i). As expected, after neutral encoding (offline 1), a higher percentage of the neutral ensemble was participating in these burst events compared with the remaining ensemble in both the low- and high-shock groups (Fig. 3c). After aversive encoding (offline 2), both groups again showed preferential participation of the aversive ensemble that was most recently active (Fig. 3d), as well as of the overlap ensemble that was previously active during both learning events (Fig. 3e). However, only in the high-shock group (and not the low-shock group), the neutral ensemble preferentially participated in these burst events as well (Fig. 3f), suggesting that a strong aversive experience drove the recruitment of the neutral ensemble into these burst events.

## Co-bursting of the overlap and neutral ensemble

Since after a high shock (during offline 2), the neutral, aversive and overlap ensembles participated in burst events (Fig. 3d–f), we next investigated whether the ensembles co-participated within the same bursts (that is, co-bursting), or whether they participated separately in different bursts. Different ensembles co-bursting could be a mechanism that integrates the neutral and aversive memory representations. Ensemble co-bursting could integrate different memories through plasticity mechanisms such as Hebbian plasticity^41^ or behavioural timescale synaptic plasticity^35^, which has been proposed to support the formation and stabilization of place fields in hippocampal neurons. Previous work has shown that aversive learning drives increased co-activity of hippocampal neurons thought to underlie the stable representation of a context memory^42^, that co-activity relationships among hippocampal neurons can distinguish between contexts^43^ and that ensembles that are highly co-active during an offline period after learning are more likely to be reactivated during memory recall than non-co-active neurons^11^. These studies suggest that co-activity of hippocampal neurons is important for storing and expressing memories.

**Fig. 4:**
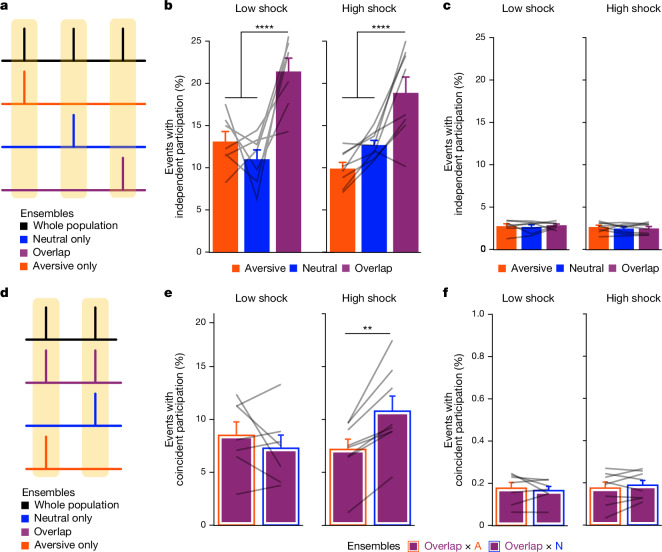
A strong aversive experience drives co-bursting of the overlap ensemble with the neural ensemble. **a**, Representation of the quantification of independent ensemble participation during burst versus non-burst periods. **b**, During burst periods, the overlap ensemble participated independently in more bursts than the aversive ensemble (*t*_14_ = 7.95, *P* = 0.000002) and more than the neutral ensemble (*t*_14_ = 5.59, *P* = 0.0001) but there was no difference in participation across low- versus high-shock mice (*F*_1,13_ = 1.43, *P* = 0.25) and no interaction (*F*_2,26_ = 2.49, *P* = 0.10). *n* = 7 (low-shock) and *n* = 8 (high-shock) mice. **c**, During non-burst periods, there was no difference in participation across ensembles (*F*_2,26_ = 0.38, *P* = 0.69) or between low- and high-shock mice (*F*_1,13_ = 0.73, *P* = 0.41), and no interaction (*F*_2,26_ = 0.36, *P* = 0.70). *n* = 7 (low-shock) and *n* = 8 (high-shock) mice. **d**, Representation of the quantification of ensemble co-participation during burst versus non-bursting periods. **e**, During burst periods, there was a significant interaction between ensemble combination and low- versus high-shock (*F*_1,13_ = 12.2, *P* = 0.004). Overlap ensemble preferentially co-participated with the neutral ensemble (N) rather than with the aversive ensemble (A) (*t*_7_ = 4.95, *P* = 0.003), whereas in the low-shock group, there was no difference in overlap ensemble participation with the neutral and aversive ensembles (*t*_6_ = 0.99, *P* = 0.36). *n* = 7 (low-shock) and *n* = 8 (high-shock) mice. **f**, During non-burst periods, there was no difference in co-participation between ensembles (*F*_1,13_ = 0.027, *P* = 0.87) or between low- and high-shock (*F*_1,13_ = 0.11, *P* = 0.74), and there was no interaction (*F*_1,13_ = 1.11, *P* = 0.31). *n* = 7 (low-shock) and *n* = 8 (high-shock) mice. Error bars indicate s.e.m.

To examine whether the neutral, aversive and overlap ensembles were co-bursting during offline 2, we measured the percentage of total burst events that each ensemble participated in, independently of each other (Fig. 4a), and the percentage that the ensembles co-participated in (Fig. 4d). Previously, we found that the overlap cells (those that are active during both neutral and aversive encoding) were also highly active during the offline period (Fig. 3e and Extended Data Fig. 4f). Highly active neurons have been proposed to form a hub-like population of neurons that may orchestrate the activity of other neurons in a network^44^. These highly active neurons could therefore be organizing and driving the activity of other hippocampal neurons during this offline period. Thus, we hypothesized that the co-bursting of the highly active overlap ensemble and the neutral ensemble would be enhanced after a strong aversive experience.

**Extended Data Fig. 5:**
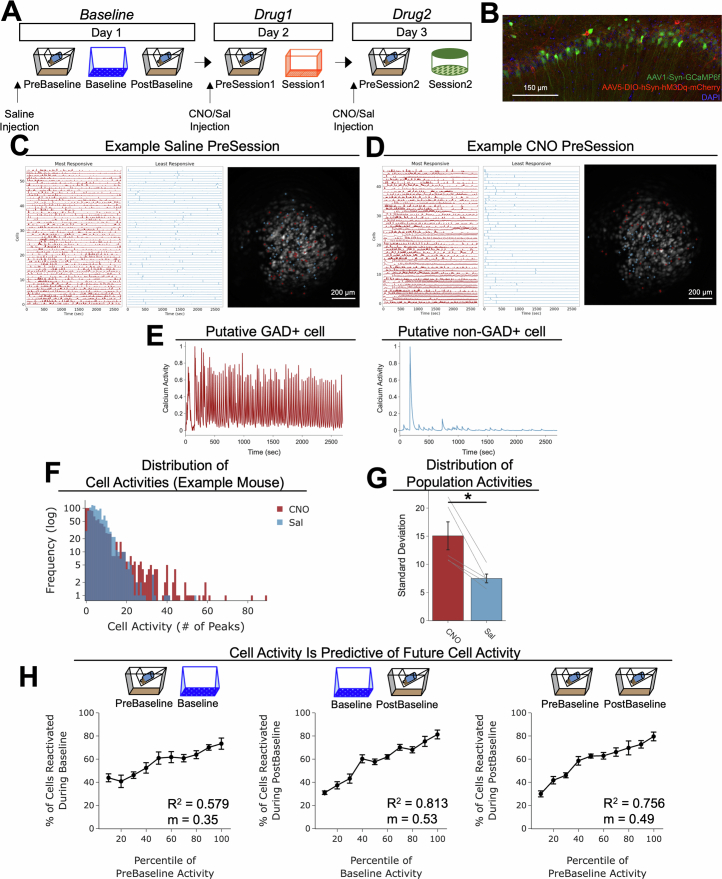
Characterization of inhibitory neuron tagging approach (chemotagging) to tag inhibitory neurons in vivo using Miniscope calcium imaging. A) Schematic of calcium imaging experiment to test whether GAD+ cells could be robustly activated with hM3Dq receptor activation in inhibitory neurons. *Gad2*-cre mice were injected with a virus cocktail of AAV1-Syn-GCaMP6f and AAV5-DIO-hSyn-hM3Dq-mCherry into dorsal CA1 and had a lens implanted above CA1. After baseplating, recovery, and habituation, mice were injected with saline and placed in their homecage for a recording (PreBaseline), followed by exposure to a novel environment (Baseline) and then another homecage recording (PostBaseline). The following day, half the mice were administered CNO and the other half saline and were placed in their homecage for a recording (PreSession1), followed by exposure to a second novel context (Session1). The following day, mice were administered with the drug they did not receive the previous day (saline or CNO) and placed in their homecage for a recording (PreSession2), followed by exposure to a third novel context (Session2). This experiment was run one time. B) Example histology of CA1 of mice in the experiment. Green represents AAV1-Syn-GCaMP6f, red represents AAV5-DIO-hSyn-hM3Dq-mCherry, and blue represents DAPI. C) Example saline PreSession. Each row represents calcium activity of a neuron, of the top 5% of most highly active cells (in red) and the bottom 5% of most lowly active cells (in blue) during the saline PreSession. On the right is a maximum intensity projection demonstrating all the cells, with red and blue crosses representing the centres of mass of the most and least active cells (respectively) from the calcium activities on the left. D) As in Extended Data Fig. 5c, but of an example CNO PreSession. Here, it is apparent that the most active cells become highly active 5−10 min after the session begins, while the most lowly active cells become inactive 5−10 min after the session begins. E) Left: a representative cell that had heightened activity during the CNO PreSession. Right: a representative cell that had inhibited activity during the CNO PreSession. These two examples represent two extremes of cells that became highly active or inhibited. Cells highly responsive to CNO suggested that they may be putative inhibitory (i.e., GAD+) neurons, whereas cells not highly responsive to CNO suggested that they may be putative non-inhibitory (i.e., GAD-) neurons. F) Example distribution of calcium activities after saline vs CNO administration in the same mouse. In this example, it is apparent that CNO widens the distribution of cell activities, consistent with activation of inhibitory neurons and inhibition of excitatory neurons. G) Quantification of the standard deviation of activity across the population after CNO vs saline. After mice received CNO, the distribution of their population activity had a larger standard deviation, consistent with the idea that some cells become very active and others become very inactive, compared with administration of saline (*t*_*4*_ = *15.04, p* = *0.018*) (*n* = *5 mice*). H) Quantification of how well the levels of cell activity at one point predict cell activity at a later timepoint. Left: The level of cell activity during the PreBaseline period is related to cell activity during Baseline (R^2^ = 0.579, slope = 0.35). Middle: Cell activity during Baseline is highly predictive of cell activity during PostBaseline (R^2^ = 0.813, slope = 0.53), as in Extended Data Fig. 3j. Right: Cell activity during PreBaseline is predictive of cell activity during PostBaseline (R^2^ = 0.756, slope = 0.49) (*n* = *5 mice*).

**Extended Data Fig. 6:**
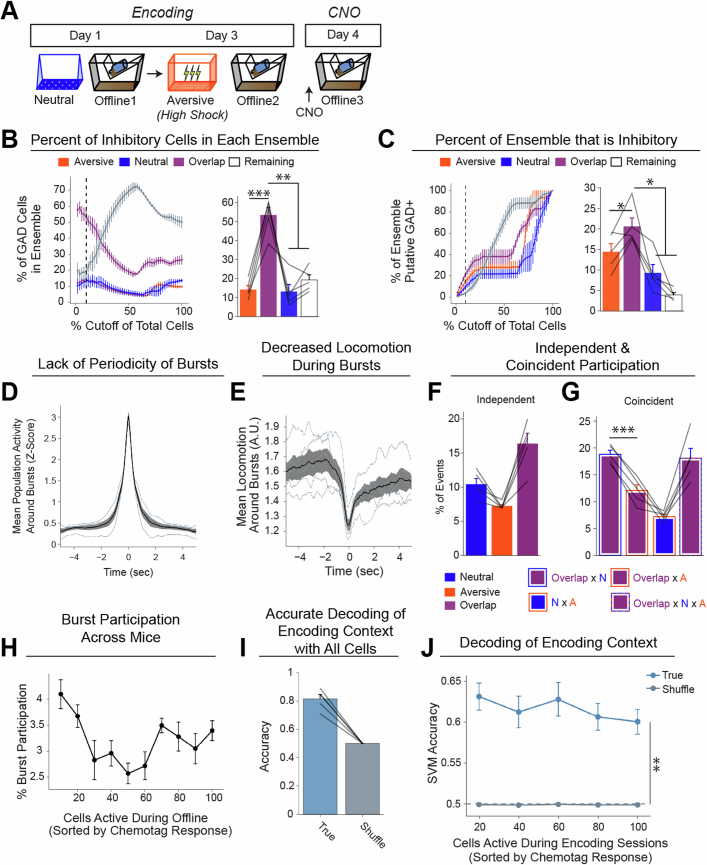
The Overlap ensemble comprises the largest fraction of inhibitory neurons. A) Schematic of calcium imaging experiment to test the breakdown of inhibitory neurons across the ensembles. *Gad2*-cre mice were injected with a virus cocktail of AAV1-Syn-GCaMP6f and AAV5-DIO-hSyn-hM3Dq-mCherry into dorsal CA1 and had a lens implanted above CA1. After baseplating, recovery, and habituation, mice underwent Neutral encoding, followed by high-shock Aversive encoding two days later. After each encoding session, mice underwent a 1 hr offline recording. A day after Aversive encoding, mice were injected with CNO to identify the putative GAD+ cells while recording calcium activity from all neurons (see Extended Data Fig. 5). Then, these cells were cross-registered back to the neurons active during the offline period the day before. This allowed us to ask, of the ensembles recorded during the offline period, what fraction of each ensemble was made up by inhibitory neurons. This experiment was run one time. B) Here, we computed the percent of cells that were putative inhibitory neurons in each ensemble. We sorted the cells recorded during Offline 2 based on how highly active they were in response to CNO administration on CNO day (from most highly active cells on CNO day to least highly active; see Methods for details on how this was computed). During Offline 2, we asked what fraction of each ensemble (i.e., Aversive, Neutral, Overlap, Remaining) made up the inhibitory neurons. Rather than specifying an a priori threshold for what fraction of recorded cells would comprise the inhibitory neuron population, we parametrically varied the threshold for what fraction of the population was made up of putative inhibitory neurons and computed the fraction of each ensemble that made up this population at each threshold. The line graph (left) represents the parametrically varied thresholds along the x-axis (i.e., % Cutoff of Total Cells), and the y-axis represents the fraction of each ensemble at each threshold cutoff. Anatomical data have suggested that inhibitory neurons make up about 10% of the total number of neurons in the pyramidal layer of CA1 (which is the region we recorded from) (Bezaire & Soltesz, 2013). Thus, we extracted the 10% mark from the line graph (represented by the black dashed line) and compared the fractions at this cutoff (right bar graph). Here, the Overlap ensemble comprised a larger fraction of the inhibitory neuron population than any of the other ensembles (*F*_*3,12*_ = *26.17, p* = *0.000015*) (*n* = *5 mice*). Notably, this effect was apparent in the line graph not only at a 10% cutoff but at neighbouring cutoffs as well. C) Here, we asked a similar question as in Extended Data Fig. 6b, but instead asking what fraction of inhibitory neurons made up each ensemble. In this case, the number of cells was a fraction of the total ensemble size. In the line graph (left), we again parametrically varied the fraction of cells that were putative inhibitory neurons along the x-axis, and asked what fraction of each ensemble was comprised of inhibitory neurons. Again, we took the 10% mark—based on anatomical data—and compared the fraction of inhibitory neurons that made up each ensemble. Similar to in Extended Data Fig. 6b, the Overlap ensemble was composed more of inhibitory neurons than the other ensembles were (*F*_*3,12*_ = *15.29, p* = *0.0002*) (*n* = *5 mice*). Collectively, this suggests that the Overlap ensemble is enriched in inhibitory neurons. D) Lack of periodicity of bursts during the offline period, as in Extended Data Fig. 3p,q (*n* = *5 mice*). E) Decrease in locomotion around bursts during the offline period, as in Fig. 2d,e (*n* = *5 mice*). F) Independent ensemble participation, as in Fig. 4b. Overlap ensemble participation is higher than Aversive participation (*t*_*4*_ = *6.1, p* = *0.01*) and is trending to be higher than Neutral participation (*t*_*4*_ = *2.55, p* = *0.063*). Neutral participation is higher than Aversive participation (*t*_*4*_ = *3.55, p* = *0.036*) (*n* = *5 mice*). G) Ensemble coincident participation, as in Fig. 4e and Extended Data Fig. 4l. Overlap x Neutral participation is higher than Overlap x Aversive (*F*_*3,12*_ = *18.99, p* = *0.0077*; *t*_*4*_ = *12.17, p* = *0.0009*), replicating the previous result in Fig. 4e (*n* = *5 mice*). H) Ensemble participation in Offline 2 bursts, as a function of the cell’s response during Inhibitory Tag, by each 5% of cells. Cells that were most active during Inhibitory Tag (leftmost points) participated more frequently in bursts than cells that responded less during Inhibitory Tag (*F*_*9,36*_ = *7.57, p* = *0.000004*) (*n* = *5 mice*). I) SVM decoding of Neutral vs Aversive encoding context using cells active during both Neutral and Aversive encoding. Accuracy of decoding is significantly higher than shuffled controls (*t*_*4*_ = *10.04, p* = *0.0006*) (*n* = *5 mice*). J) SVM decoding of Neutral vs Aversive encoding is no different when using 20% of cells, based on the cells’ response during Inhibitory Tag (*F*_*4,16*_ = *1.50, p* = *0.28*). This suggests that the putative inhibitory neurons hold no more or less predictive power than the rest of the population. All decoders performed better than shuffled controls (*F*_*1,4*_ = *64.13, p* = *0.001*). There is no interaction (*F*_*4,16*_ = *1.44, p* = *0.29*) (*n* = *5 mice*).

We found that during burst events, the overlap ensemble participated independently more frequently than the neutral and aversive ensembles, but there was no difference between the low- and high-shock groups (Fig. 4b). Notably, during non-burst periods, independent ensemble participation was not different between any of the ensembles or groups (Fig. 4c). We next measured co-bursting (that is, co-participation in burst events) of the overlap ensemble with the neutral ensemble or with the aversive ensemble (Fig. 4d). We found that in the high-shock group, the overlap ensemble co-bursted more with the neutral ensemble (overlap × neutral) than with the aversive ensemble (overlap × aversive) (Fig. 4e). However, in the low-shock group, there was no difference between co-bursting of the overlap ensemble with the neutral or aversive ensembles (Fig. 4e and Extended Data Fig. 4l–o). Importantly, there were no differences in ensemble co-bursting between the low- and high-shock groups during non-burst periods (Fig. 4f). These results suggest that after a strong aversive experience, the overlap ensemble was preferentially co-bursting with the neutral ensemble, confined to periods of synchronous hippocampal activity. To confirm this, we used cross-correlations as another measure of co-activity to measure how co-active the overlap ensemble was with the neutral and the aversive ensembles. Indeed, only in the high-shock group, the overlap ensemble was preferentially correlated with the neutral ensemble compared with the aversive ensemble during the offline period (Extended Data Fig. 4k). Because the overlap ensemble was preferentially co-bursting with the neutral ensemble in the high-shock group, we examined whether the overlap ensemble, which consisted of highly active cells (Figs. 3e and 4b and Extended Data Fig. 4f), could represent a hub-like population of neurons that could help to orchestrate the activities of neighbouring neurons. Inhibitory neurons in the hippocampus are known to make thousands of synaptic contacts with neighbouring neurons^45^ and, as a result, have an outsized influence on the activities of the local network. Moreover, subclasses of inhibitory neurons are known to fire at specific times relative to sharp wave ripples^46^ and their oscillation time-locked activity is thought to gate which neurons reactivate during brief reactivation events^44,47^. Thus, we tested whether the overlap ensemble was highly composed of inhibitory neurons. To do this, we developed an approach to record pan-neuronal calcium imaging and identify inhibitory neurons using cell-type specific chemogenetics post hoc, which we termed chemotagging (Methods and Extended Data Fig. 5). Using this approach, we measured the inhibitory/excitatory neuron composition of the four ensembles recorded during offline 2 after aversive encoding (Extended Data Fig. 6). Indeed, we found that the overlap ensemble was enriched in putative inhibitory neurons (Extended Data Fig. 6b,c). In this experiment, we also replicated the co-reactivation of the overlap ensemble with the neutral ensemble during offline 2, as in Fig. 4e (Extended Data Fig. 6g).

**Extended Data Fig. 7:**
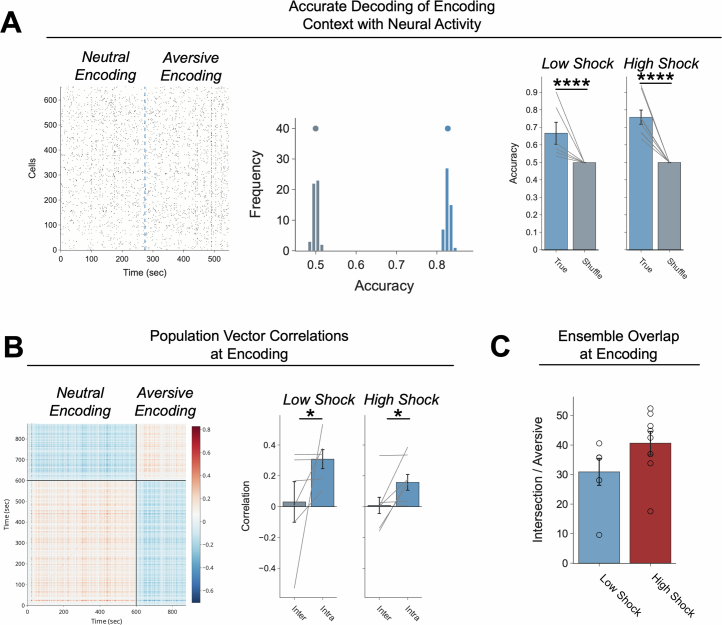
Neutral and Aversive contexts are discriminable at encoding. A) Left: Example raster of activity of cells during Neutral and Aversive encoding. Cells that were cross-registered during both sessions were aligned, concatenated, and labelled with the context they were associated with. Support vector machines (SVMs) were trained on this activity (see Methods for details). Middle: Example distributions of accuracy using the true data (in blue) and using shuffled label controls (in grey). The dots above the distributions represent the mean accuracy of the distribution. Right: Quantification of accuracy between True vs Shuffle in low- and high-shock mice. Accuracy of the True data was significantly higher than Shuffle in both low- and high-shock groups (*F*_*1,12*_ = *38.49, p* = *0.000046*), and there was no difference in low- and high-shock mice (*F*_*1,12*_ = *0.015, p* = *0.22*) and no interaction (*F*_*1,12*_ = *0.014, p* = *0.23*) (*low-shock, n* = *6 mice; high-shock, n* = *8 mice*). B) Left: Example of population vector correlations within and between Neutral and Aversive encoding. Cells across sessions were again aligned and concatenated, as in Extended Data Fig. 7a. Then moment-to-moment correlation matrices were constructed to compare population activity within (intra) and between (inter) encoding sessions. Right: Quantification of population vector correlations within and between sessions. Intra-session correlations were higher than Inter-session correlations (*F*_*1,12*_ = *6.74, p* = *0.02*). There was no effect of Amplitude (*F*_*1,12*_ = *1.50, p* = *0.24*) or Interaction (*F*_*1,12*_ = *0.65, p* = *0.44*). Moreover, Intra-session correlations were significantly greater than 0 in low-shock (*t*_*5*_ = *5.00, p* = *0.016*) and high-shock (*t*_*7*_ = *3.08, p* = *0.036*), whereas Inter-session correlations were not significantly greater than 0 in low-shock (*t*_*5*_ = *0.23, p* = *0.88*) or high-shock (*t*_*7*_ = *0.15, p* = *0.88*). (*low-shock, n* = *6 mice; high-shock, n* = *8 mice*). C) Ensemble overlap between Neutral and Aversive encoding in low- vs high-shock mice. There was no difference in ensemble overlap between Neutral and Aversive encoding in low- vs high-shock mice (*t*_*11.43*_*, p* = *0.13*) (*low-shock, n* = *6 mice; high-shock, n* = *8 mice*).

**Extended Data Fig. 8:**
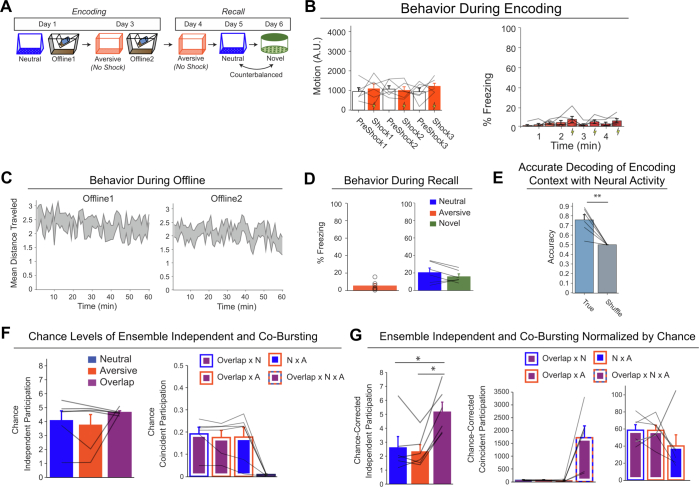
No Shock control mice do not display co-bursting between the Overlap and Neutral ensembles. A) Schematic of calcium imaging experiment performing retrospective memory-linking with no-shock during Aversive encoding. Mice underwent Neutral encoding followed by Aversive encoding two days later, during which they received 0 mA shocks (i.e., no shocks). In the subsequent three days, mice were tested in the Aversive context, followed by Neutral and Novel recall, counterbalanced. This experiment was run one time. B) Behaviour during Aversive encoding. Left: We measured locomotion at the same timepoints as in Extended Data Fig. 4b, where low- and high-shock mice received footshocks. As expected, mice did not display a change in locomotion during these timepoints, in contrast to low- and high-shock mice in Extended Data Fig. 4b. Right: As expected, mice did not display an increase in freezing across the Aversive encoding session, in contrast to low- and high-shock mice in Extended Data Fig. 4a. C) Locomotion during the offline periods. Locomotion gradually decreased throughout the offline period during both Offline 1 and Offline 2 (*n* = *6 mice*). D) Freezing during recall. Left: Mice froze minimal levels during Aversive recall. Right: Mice froze no differently in Neutral vs Novel recall (*t*_*5*_ = *1.52, p* = *0.19*) (*n* = *6 mice*). E) SVM performance during encoding. An SVM predicted Neutral vs Aversive encoding context more accurately than shuffled controls, as in Extended Data Fig. 7a (*t*_*5*_ = *4.77, p* = *0.005*) (*n* = *6 mice*). F) Chance levels of ensemble independent and co-bursting during the offline period. Left: there were no differences in chance levels of ensemble independent participation (*F*_*2,10*_ = *1.07, p* = *0.38*). Right: there were no differences in chance levels of co-bursting of any two ensembles, and co-bursting of two ensembles was higher than co-bursting of all three (*F*_*3,15*_ = *20.99, p* = *0.000013*) (*n* = *6 mice*). G) Ensemble independent and co-bursting normalized by chance during the offline period. Left: independent participation of the three ensembles. Overlap ensemble independent participation was higher than either Aversive (*t*_*5*_ = *6.31, p* = *0.004*) or Neutral (*t*_*5*_ = *3.8, p* = *0.019*) independent participation. Middle: Ensemble co-bursting of all combinations. Right: Replotting of co-bursting between only two ensembles. There was no difference in co-bursting between the ensemble pairs (*F*_*2,10*_ = *1.29, p* = *0.32*) (*n* = *6 mice*).

To test whether the neural representations of the neutral and aversive contexts were already linked during encoding, we compared neural activity during neutral and aversive encoding in low- and high-shock mice. We found that the neural activity patterns for neutral and aversive contexts were highly discriminable. There were no differences between the low- and high-shock groups during encoding (Extended Data Fig. 7). Finally, we examined whether we would observe ensemble co-bursting if we removed the negative emotional valence. To do this, we repeated the retrospective memory-linking calcium imaging experiment; however, when we would typically conduct aversive encoding, we administered no foot shocks (that is, the no-shock group; Extended Data Fig. 8). In this group, similar to in the low-shock group, we found that neural activity during encoding of the two contexts was highly discriminable (Extended Data Fig. 8e), that the co-bursting of the overlap and neutral ensembles was not different from the co-bursting of the overlap and aversive ensembles during the offline period (Extended Data Fig. 8f,g), and that these mice showed no differences in freezing in the neutral versus novel contexts during recall (Extended Data Fig. 8d). Collectively, these results suggest that a strong aversive experience increases the co-bursting of the overlap ensemble with the neutral ensemble, providing a circuit mechanism to link fear of the recent aversive experience with the past neutral memory.

## Co-reactivation occurs more during wake

**Fig. 5:**
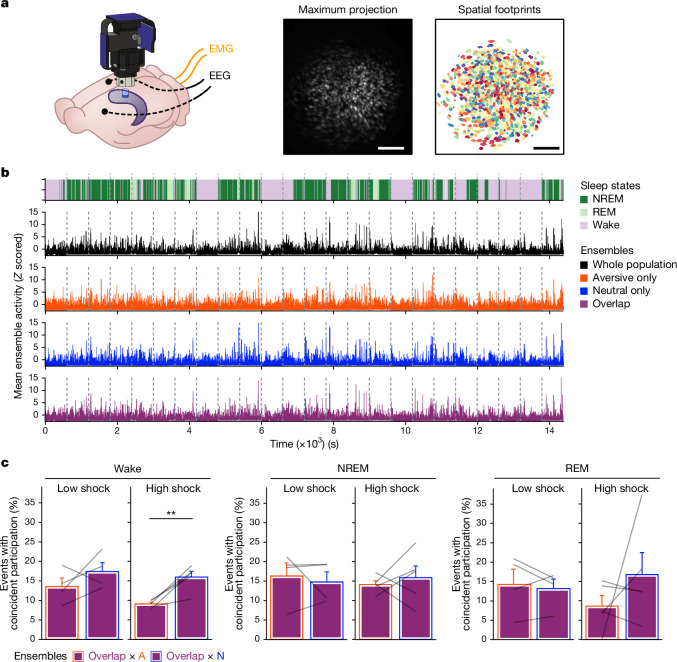
Co-reactivation between the overlap and neutral ensembles occurs during more wake than during sleep. **a**, Schematic of the GRIN lens and electrode implants used for this experiment (left). Mice were injected with AAV1-Syn-GCaMP6f in the dorsal CA1. Then, 2 weeks later, the mice were implanted with a lens above the injection site, with two EEG electrodes and two EMG electrodes. Next, 2 weeks after this, the mice were implanted with a baseplate for Miniscope calcium imaging. Middle, maximum-intensity projection of an example mouse across one recording session, imaged using a Miniscope. Right, the spatial footprints of all recorded cells during that session, randomly colour coded. Each mouse was run one at a time for this experiment. Scale bars, 200 μm. **b**, Example of 24 concatenated calcium imaging offline sessions. Top, the sleep state across all the calcium imaging recordings. Bottom, the whole-population mean activity, the aversive ensemble mean activity, the overlap ensemble mean activity and the neural ensemble mean activity. The dotted grey lines represent the boundaries between each offline recording. **c**, Ensemble co-bursting across sleep states. Left, wake high-shock mice had higher co-bursting of overlap × neutral than overlap × aversive (*t*_4_ = 4.94, *P* = 0.016) while low-shock mice had no difference in co-bursting between these ensembles (*t*_3_ = 1.20, *P* = 0.32). Middle, for NREM, there was no difference in high-shock (*t*_4_ = 0.53, *P* = 0.66) or low-shock (*t*_3_ = −0.49, *P* = 0.66) co-bursting. Right, for REM, there was no difference in high-shock (*t*_4_ = 1.04, *P* = 0.63) or low-shock (*t*_3_ = −0.53, *P* = 0.63) co-bursting. *n* = 4 (low-shock) and *n* = 5 (high-shock) mice. Error bars indicate s.e.m.

**Extended Data Fig. 9:**
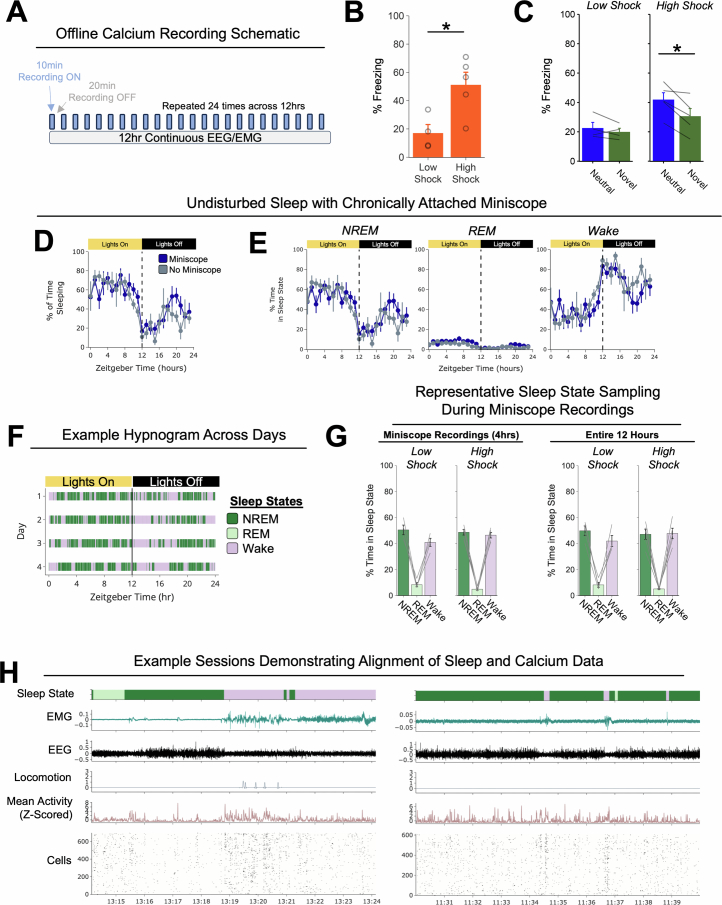
Validation of simultaneous calcium imaging and EEG/EMG to measure ensemble reactivation across sleep states. A) Schematic representing the protocol for calcium imaging and EEG/EMG recordings during the offline period. EEG and EMG were recorded continuously throughout the offline period. To avoid photobleaching, calcium imaging was done intermittently: calcium was recorded for 10 min, followed by 20 min of no recording, repeated 24 times, for 4 hrs worth of calcium recordings across a 12 hr period. Mice underwent the retrospective memory-linking behavioural paradigm as in Fig. 1d, with calcium imaging recordings during Neutral and Aversive encoding, as well as during Aversive, Neutral, and Novel recall. During Offline 1 and Offline 2, in contrast to in Fig. 1d, these mice underwent the recording scheme described above. B) Freezing during Aversive recall. High-shock mice froze more during Aversive recall than low-shock mice (*t*_*7*_ = *8.99, p* = *0.020*) (*low-shock, n* = *4 mice; high-shock, n* = *5 mice*). C) Freezing during Neutral vs Novel recall. High-shock mice froze more during Neutral vs Novel recall (*t*_*4*_ = *4.02, p* = *0.03*), whereas low-shock mice froze no differently in Neutral vs Novel recall (*t*_*3*_ = *1.08, p* = *0.36*) (*low-shock, n* = *4 mice; high-shock, n* = *5 mice*). D) Mice wearing a Miniscope chronically throughout the experiment display no differences in sleep duration across the 24 hr sleep/wake cycle, compared to mice with no Miniscope implant (*F*_*1,10*_ = *0.54, p* = *0.48*) (*Miniscope, n* = *8 mice; No Miniscope, n* = *4 mice*). E) Same as Extended Data Fig. 9d but broken up by sleep state. Mice wearing a Miniscope chronically show no differences in time spent in each sleep state. F) Example hypnogram demonstrating that mice display normal patterns of sleep, with more bouts of sleep during their Lights On period. G) The calcium imaging recording scheme in Extended Data Fig. 9a reliably captures the fractions of time that mice spend in each sleep/wake state. Left: Amount of time spent in each sleep state as captured during the Miniscope recordings across a 12 hr period (4hrs of total calcium recording time). Right: Amount of time spent in each sleep state across the entire 12hrs of the offline period (*low-shock, n* = *4 mice; high-shock, n* = *5 mice*). H) Example sessions demonstrating alignment of sleep and calcium data. Left: Example session where mouse awakens halfway throughout the recording. Right: Example session where mouse is mostly asleep but has three brief arousals.

**Extended Data Fig. 10:**
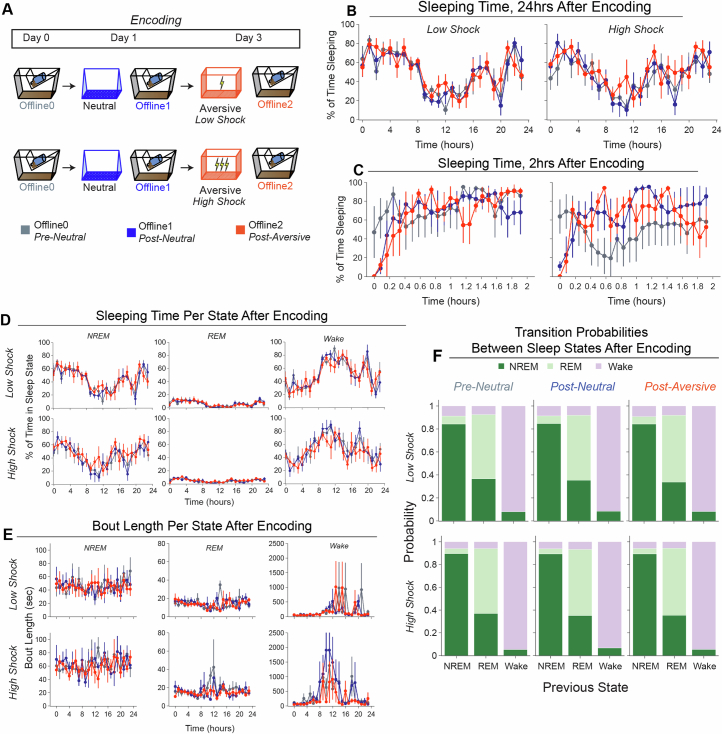
Sleep structure is not modified by Neutral or Aversive encoding. A) Schematic of experiment comparing sleep patterns before and after each encoding experience. Mice had their EEG/EMG recorded for the 24 h prior to the start of the first Neutral encoding experience, and for the 24 hrs following Neutral encoding and after Aversive encoding. B) Mice display no gross change in sleep duration across days (Pre-Neutral vs Post-Neutral vs Post-Aversive), or in low- vs high-shock mice (*Low Shock, n* = *4 mice; high-hock, n* = *5 mice*). C) When we zoom into the first 2hrs after encoding, we see that mice are awake for longer for about the first 30 min after encoding, after which sleep patterns return to pre-experiment levels (*low-shock, n* = *4 mice; high-shock, n* = *5 mice*). D) Same as Extended Data Fig. 10b but broken up for time spent in each sleep state. E) Mice display no differences in bout length of each sleep state across days (Pre-Neutral vs Post-Neutral vs Post-Aversive), or in low- vs high-shock mice (*low-shock, n* = *4 mice; high-shock, n* = *5 mice*). F) Mice display no differences in transition probabilities between sleep states across days (Pre-Neutral vs Post-Neutral vs Post-Aversive), or in low- vs high-shock mice (*low-shock, n* = *4 mice; high-shock, n* = *5 mice*).

Ensemble reactivation has previously been observed to occur during non-rapid-eye-movement (NREM) sleep^17^, REM sleep^8^ and wake periods^48^. This reactivation has been proposed to support memory consolidation, among other memory and decision-making functions^13–15^. Thus, we next examined whether ensemble co-reactivation occurred preferentially during a specific sleep/wake state to support retrospective memory linking. To investigate this, we performed simultaneous calcium imaging and electroencephalogram (EEG) and electromyography (EMG) recordings in mice that underwent the low- and high-shock retrospective memory-linking procedure, as in Fig. 1d (Fig. 5 and Extended Data Fig. 9). Here, mice were attached to a Miniscope chronically throughout the approximately 2 week experiment along with a chronically implanted EEG/EMG telemetry device (Methods). This enabled us to record across a 12 h offline period rather than the 1 h offline recordings in Figs. 2–4. We first validated that mice could wear the Miniscope without disruptions to their sleep (Extended Data Fig. 9d–f). We confirmed that our calcium recording scheme reliably captured all sleep states (Extended Data Fig. 9g). We also replicated the retrospective memory-linking behaviour (Extended Data Fig. 9b,c). We next examined whether sleep patterns were altered after neutral or aversive encoding. There were no differences in sleep features (total sleep time, time in each sleep/wake state, bout length or transitions between sleep states) after the neutral or aversive encoding compared to pre-experiment sleep (Extended Data Fig. 10). We then measured ensemble co-bursting during the offline periods as in Fig. 4, but here separated by sleep/wake states (Fig. 5b). We found that as in Fig. 4e, in the high-shock group, the overlap ensemble preferentially co-bursted with the neutral ensemble more than with the aversive ensemble during wake. This effect was absent in the low-shock group, and it was absent during NREM and REM sleep in both of the groups (Fig. 5c). This result of co-bursting during wake, along with our previous finding that population bursts coincided with the animal briefly pausing its locomotion (Fig. 2d,e and Extended Data Fig. 6e), suggest that this preferential ensemble co-reactivation occurs during brief periods of quiet wake.

## Ensemble co-reactivation in neutral context recall

Finally, we examined how hippocampal ensemble reactivation contributed to the freezing observed during recall in the neutral context after a high shock and not after a low shock (Fig. 1f and Extended Data Figs. 1e and 9c). Previously, we found that after aversive encoding during offline 2, the overlap ensemble co-reactivated with the neutral ensemble in the high-shock group (Fig. 4e and Extended Data Figs. 4k–o and 6g), perhaps forming an integrated ensemble of neurons that is more likely to fire together in the future. If this were the case, during neutral recall, we expected that the neutral ensemble would be reactivated to recall the neutral context. We predicted that the reactivation of the neutral ensemble might trigger the reactivation of the overlap ensemble, perhaps through a process of pattern completion^49^, thereby driving freezing in the neutral context. Importantly, we did not expect this to occur in low-shock mice during neutral recall, where neutral and overlap ensemble co-reactivation was observed at a lower level, or in high-shock mice during novel-context exposure, as fear did not selectively spread to the novel context (Fig. 1f).

**Fig. 6:**
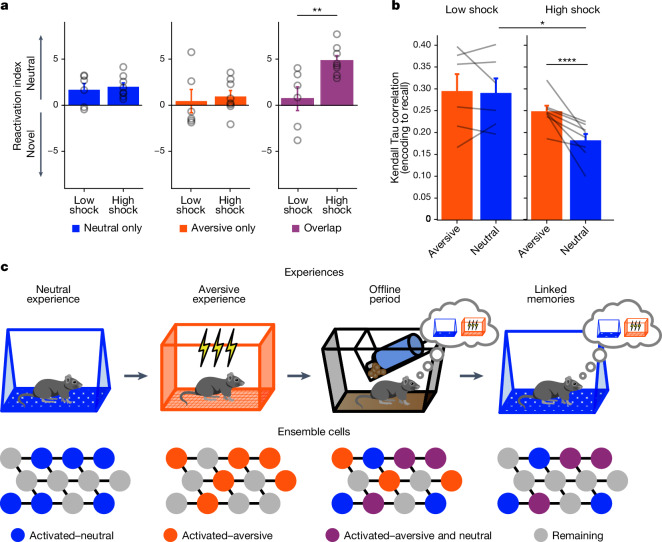
A strong aversive experience drives ensemble co-reactivation during neutral context recall. **a**, Ensemble reactivation during neutral versus novel recall. The reactivation index was computed as the difference in ensemble overlap between the neutral versus novel contexts (that is, reactivation during neutral − reactivation during novel; Methods) (ensemble overlap percentages are shown in Extended Data Fig. 4q–s). Left, there was no difference between the low- and high-shock groups in the reactivation index of the neutral ensemble (*t*_12_ = 0.42, *P* = 0.68). Middle, there was no difference in the aversive ensemble (*t*_12_ = 0.38, *P* = 0.71). Right, there was a significant difference in the overlap ensemble (*t*_12_ = 3.2, *P* = 0.007). **b**, In high-shock mice, population activity patterns in the neutral context changed significantly from neutral encoding to neutral recall (amplitude: *F*_1,12_ = 5.65; session pair: *F*_1,12_ = 10.42; amplitude × session pair: *F*_1,12_ = 6.22). During neutral recall in high-shock mice, population activity vectors were less correlated with the average neutral encoding population vector than aversive recall activity was with the average aversive encoding population vector (*t*_7_ = 4.10, *P* = 0.009). Neutral encoding-to-recall correlations were also lower in high- versus low-shock mice (*t*_6,92_ = 2.98, *P* = 0.042). Aversive encoding-to-recall correlations were no different in the high- versus low-shock mice (*t*_6,11_ = 1.13, *P* = 0.30). In low-shock mice, neutral and aversive encoding-to-recall correlations were no different (*t*_5_ = 0.23, *P* = 0.83). *n* = 6 (low-shock) and *n* = 8 (high-shock) mice. **c**, Single experiences are encoded by neurons that are highly active during learning. During the offline period after a strong aversive experience, not only is the aversive ensemble reactivated to consolidate that memory, but a past neutral memory ensemble is also reactivated, linking the aversive and neutral memories. During recall of the neutral memory, the linked memory ensemble is reactivated to drive fear in the neutral context. Error bars indicate s.e.m.

Of the cells that were active during recall of the neutral context or exposure to a novel context, we investigated the percentage that was previously active during neutral encoding, aversive encoding or both (Fig. 6a). As expected, cells exclusively active during neutral encoding and not aversive encoding were reactivated more during neutral recall than during novel-context exposure (Fig. 6a (left) and Extended Data Fig. 4q). This was the case in both the low-shock and high-shock groups, suggesting a stable and selective neural population representing the neutral context memory. The cells exclusively active during aversive encoding were not selectively reactivated during the neutral or novel contexts in either group (Fig. 6a (middle) and Extended Data Fig. 4r). Importantly, the cells that were active during both neutral and aversive encoding (overlap ensemble) were reactivated more during neutral recall than novel-context exposure in the high-shock but not the low-shock group (Fig. 6a (right) and Extended Data Fig. 4s). This suggests that after ensemble co-reactivation of the neutral and overlap ensembles during the offline period after a high shock, these ensembles were more likely to reactivate during neutral recall.

The strong aversive experience prompted an ensemble from days ago to be reactivated offline. During subsequent neutral recall, mice exhibited increased freezing despite never having been shocked in that context. If this offline reactivation of the neutral ensemble was indeed modifying the neutral memory representation, we hypothesized that during neutral recall, the activity patterns observed would be different from those during neutral encoding in high-shock mice compared with in low-shock mice, and perhaps compared with the change observed from aversive encoding to aversive recall. To measure the similarity between activity patterns during encoding and recall, we computed a mean population activity vector during neutral encoding and correlated it with 30 s population vectors across neutral recall (Methods). We repeated this for aversive encoding and correlated it with activity patterns during aversive recall. Consistent with our hypothesis, neutral encoding-to-recall correlations were lower in high-shock mice compared with in low-shock mice (Fig. 6b), suggesting that the neutral memory representation was significantly altered from encoding to recall in high-shock mice. In high-shock mice, the neutral encoding-to-recall correlations were also lower than aversive encoding-to-recall correlations (Fig. 6b), suggesting that the neutral memory representation was modified more than the aversive memory representation. These results collectively suggest that a strong aversive experience drove the overlap and neutral ensembles to co-reactivate during the offline period, altering the neutral memory representation. During neutral recall, these ensembles were again co-reactivated, leading to the increased freezing observed in the neutral context (Fig. 6c).

## Discussion

How animals dynamically update memories as they encounter new information remains a fundamental question in neuroscience^22^. Past work has shown that individual experiences are encoded by subpopulations of neurons across the brain that are highly active during learning^6,24^. After learning, the activity of these ensembles is necessary^4^ and sufficient^3,5^ for memory recall, and their reactivation during memory recall is correlated with memory recall strength^1,6^. How memories encoded across time are integrated remains a critical and unanswered question in neuroscience. The memory-allocation hypothesis suggests that neurons with high intrinsic excitability at the time of learning are likely to be allocated to a memory trace^24^. Previous studies suggest that two memories encoded within a day are likely to be linked because they share an overlapping population of highly excitable neurons during the initial learning. This shared neural ensemble links the two temporally related memories, such that the recall of one memory is more likely to trigger the recall of another memory that was encoded close in time^24,29,30,50^. While these studies have focused on how memories can be linked during the encoding process, here we demonstrate that memories are dynamically updated even days after they have been encoded and consolidated, and that this process is driven by ensemble co-reactivation during a post-learning period.

### Linking the present with the past can make predictions about the future

Whether linking memories across days is an adaptive or maladaptive process may depend on the environmental conditions. Under everyday circumstances, memories that are encoded far apart in time and that share no features in common are not typically linked. Memories must be segregated to allow proper recall of distinct memories. Notably, the hippocampus has been shown to successfully discriminate between distinct memories^49^. However, after a potentially life-threatening experience, especially one in which the source of the aversive outcome is ambiguous (as in the aversive experience used here), it could benefit an animal to link fear from that aversive experience to previous events, particularly if the event is rare and novel as also seen in conditioned taste aversion^23^. Consistent with this intuition, our results suggest that a highly emotional and salient experience drives retrospective memory linking (Fig. 1d–g and Extended Data Figs. 1c–e, 2 and 9b,c). Moreover, our results suggest that fear is more likely to be linked retrospectively to past events rather than prospectively to future events separated by days (Fig. 1a–c and Extended Data Fig. 1a,b). This is consistent with the notion that cues present before an outcome can predict that outcome. On a shorter timescale, it has been well established that when a neutral cue directly precedes a foot shock by seconds, this drives associative learning between the cue and the foot shock, leading to cue-elicited freezing^51^. However, if the cue instead occurs directly after the foot shock, the animal no longer freezes in response to cue presentation thereafter, presumably because the cue predicts the ensuing absence of the aversive event^52^. Although the difference in timescale suggests that different mechanisms are likely at play in these two scenarios, our results are consistent with the idea that cues occurring before an outcome can be interpreted as predictive cues to the animal. A recent review has also suggested that animals use retrospective cognitive maps to infer the states that precede an outcome to draw causal associations between those stimuli^53^. Our results suggest that offline periods are important for this retrospective inference (Fig. 6c).

### Offline periods promote association between memories not previously linked

Offline periods offer an opportunity for the brain to draw inferences about relationships that were not necessarily formed at the time of learning. In humans, it has been shown that an emotional experience can retrospectively enhance memory strength for previously learned neutral objects, only after a period of consolidation^32^. In that study, the participants were exposed to neutral stimuli and, 5 min later, were fear conditioned to conceptually similar stimuli. There was only an enhancement of the neutral memory if a consolidation window was imposed after fear conditioning. A separate study demonstrated that this retrospective memory enhancement coincided with increased functional hippocampal–cortical coupling and fMRI BOLD activity in the ventral tegmental area and locus coeruleus^33^. Together, these studies in human participants suggest that offline periods offer an opportunity for memories to interact and become linked.

Moreover, in mice, a recent study showed that two contexts with strongly shared geometrical features can be integrated immediately after learning (that is, 15 min after learning), whereas two contexts with subtly shared geometrical features require an offline period (that is, 1 day after learning) to drive their integration^54^. During this offline period, cortical ensemble co-reactivation drives this memory integration. Retrospective memory linking occurs through co-reactivation of the ensembles for the two memories during an offline period, probably across multiple brain regions and can be modulated by the aversiveness of the experience.

### Sleep and wake states promote distinct memory processes

Past studies have shown that ensemble reactivation occurs during both sleep (NREM and REM sleep) and wake states. Reactivation during different states has been proposed to support different memory processes. For example, classical studies demonstrated that after a salient experience, the patterns of neuronal activity that were present during learning are sequentially replayed offline, and this replay has been observed during both NREM and REM sleep^13–15^. The replay observed during sleep was proposed to support memory consolidation and, indeed, disruption of sharp-wave ripples (during which most of these replay events occur) disrupts the storage of memories such that memory recall is disrupted thereafter^19,20^. Notably, one study found that prolonging sharp-wave ripple durations benefited memory, whereas cutting them short impaired memory^21^. In addition to during sleep, it has also been observed that sharp-wave ripples and hippocampal replay occur while animals are awake, and it can occur in a forward or reverse direction^9^. This has led to the idea that different forms of replay may have different functions, from memory consolidation to planning and decision-making^13–15^, although this remains a debate^55^. Our results demonstrate that ensemble co-reactivation supporting memory integration is a phenomenon that occurs most during wake periods. More specifically, the transient population bursts during which we observed ensemble co-reactivation occurred during brief periods of quiet wake (Fig. 2d,e and Extended Data Fig. 6e). Thus, while memory consolidation is supported by ensemble reactivation during sleep, retrospective memory linking may be supported by ensemble co-reactivation during periods of wakeful quiescence.

### Ensemble co-activity supports retrospective memory linking

A long history of literature suggests that the specific timing of activity among neurons regulates whether ensembles of neurons will strengthen or weaken their connections and, therefore, be more or less likely to fire together long-term (that is, cells that fire together, wire together)^35,41,56^. Consistent with this literature, we demonstrate here that ensembles representing two distinct memories can be reactivated together during offline periods, and this co-reactivation drives the long-term integration of these populations of neurons, such that they are both more likely to be active again days later, when animals recall the past neutral memory. Critically, this co-reactivation is dependent on the population of neurons that are active during both the neutral and aversive learning experiences (that is, the overlap ensemble; Figs. 2–6). This highly active ensemble may function as a hub-like ensemble of neurons that can orchestrate the firing of other cells in the population during offline periods^44^. Inhibitory neurons in the pyramidal layer of the hippocampal CA1, which make thousands of synapses with neighbouring neurons, have the potential to exert an outsized influence on the activity of neurons in the region. For example, inhibitory neurons in the CA1 that are highly active around the onset of sharp-wave ripples are thought to gate which excitatory neurons fire during and after sharp-wave ripples^57^. Moreover, it has been shown that inhibitory neurons have a direct role in encoding aversive memories, rather than solely a role in modulating excitatory neuron activity^58^. Notably, during the offline period, the overlap ensemble included a large number of inhibitory neurons (Extended Data Fig. 6). The overlap ensemble also participated more during population bursts than the other ensembles (Fig. 4b and Extended Data Fig. 6f) and displayed specific co-firing with other memory-relevant ensembles (Figs. 4e and 5c and Extended Data Fig. 6g), consistent with a hub-like neuronal population.

### Translation implications

Finally, these results have implications for interpretation of the clinical manifestation of memory-related conditions such as post-traumatic stress disorder (PTSD). PTSD transpires from one or multiple traumatic events and is hallmarked by uncontrollable fear in non-life-threatening contexts^59^. A common form of behavioural treatment for PTSD is exposure therapy, whereby the patient is carefully re-exposed to the trauma-associated conditioned stimuli, seeking to detach the association between those stimuli and fear. In many cases, exposure therapy successfully decreases fear, but patients are often prone to relapse thereafter^60^. Our results suggest that highly salient aversive experiences can drive fear to be associated with seemingly unrelated stimuli that were not present at the time of the aversive experience, and that this scales with the perceived aversiveness of the experience (Fig. 1g). This predicts that although exposure therapy may successfully inhibit fear to the trauma stimuli, the fear from the trauma may have spread to other stimuli that were not directly targeted by the therapy. Thus, it may be useful to consider stimuli that were experienced across time that may have insidiously become linked with the trauma. Our results point to the offline period after an aversive event as a potential intervention timepoint to unlink memories separated across days.

Taken together, our results suggest that after a highly emotional and salient experience, the brain not only reactivates the recent experience to consolidate that memory, but also co-reactivates past memories from days ago. This co-reactivation of multiple experiences during a period of quiet wake integrates memories across time. This has important implications for both adaptive memory processes (such as making causal inferences) and maladaptive processes (such as overgeneralization of fear in PTSD).

## Methods

### Mice

Adult C57BL/6J wild-type male mice from Jackson Laboratories were used in all experiments except for inhibitory tagging experiments (Extended Data Figs. 5 and 6). In those experiments, *Gad2*-*cre* male mice from Jackson Laboratories (or bred in-house from Jackson Laboratories) were used. Mice ordered from Jackson arrived group-housed in cages of 4 mice per cage and were singly housed for the experiment. Mice underwent behavioural testing at 12–18 weeks of age. For experiments in which mice underwent PSAM virus injections, mice were included in the experiment if there was expression of GFP^+^ cell bodies in both the dorsal and ventral hippocampus. All experimental procedures were approved by the Icahn School of Medicine at Mount Sinai’s IACUC.

### Viral constructs

For calcium imaging experiments in Figs. 2–6 and Extended Data Figs. 3, 4 and 7–10, AAV1-Syn-GCaMP6f-WPRE-SV40 (titre, 2.8 × 10^13^ genome copies per ml) was purchased from AddGene and was diluted by 4 in sterile 1× PBS (final titre, ~7 × 10^12^ genome copies per ml). The mice had 300 nl of the diluted virus injected into the right hemisphere of the dorsal CA1. For PSAM experiments, AAV5-Syn-PSAM4-GlyR-IRES-eGFP (2.4 × 10^13^ genome copies per ml) was purchased from AddGene. Mice had the virus injected at stock titre bilaterally into the dorsal and ventral hippocampus, 300 nl per injection site. For inhibitory tagging experiments, a virus cocktail of AAV1-Syn-GCaMP6f-WPRE-SV40 (titre, 1.3 × 10^13^ genome copies per ml) and AAV5-hSyn-DIO-hM3Dq-mCherry (titre, 2.6 × 10^13^ genome copies per ml) (both purchased from AddGene) was mixed 1:1 and mice had 300 nl of this mixed virus cocktail injected into the right hemisphere of the dorsal CA1.

### Surgery

Mice were anaesthetized with 1 to 2% isoflurane for surgical procedures and placed into a stereotaxic frame (David Kopf Instruments). Eye ointment was applied to prevent desiccation, and the mice were kept on a heated pad to prevent hypothermia. Surgery was performed using aseptic technique. After surgery, carprofen (5 mg per kg) was administered every day for the following 3 days, and ampicillin (20 mg per kg) was administered every day for the next 7 days. For calcium imaging experiments, dexamethasone (0.2 mg per kg) was also administered for the following 7 days.

For PSAM experiments (Extended Data Fig. 1l–p), AAV5-Syn-PSAM4-GlyR-IRES-eGFP was injected at stock concentration. Mice had 300 nl of the virus injected bilaterally into the dorsal hippocampus (anteroposterior (AP), −2 mm; mediolateral (ML), ±1.5 mm; dorsoventral (DV), −1.5 mm) and 300 nl injected bilaterally into the ventral hippocampus (AP, −3 mm; ML, ±3.2 mm; DV, −4 mm), for a total of four injections and 1.2 μl injected per mouse, using a glass pipette and the Nanoject injector. The pipette was slowly lowered to the injection site, the virus was injected at 2 nl s^−1^ and then the pipette remained for 5 min before being removed to allow diffusion of the virus. Mice had their incision sutured after surgery and betadine was applied to the site to prevent infection.

For calcium imaging experiments in Figs. 1–4 and 6, mice underwent two serial procedures spaced 1 month apart, as described previously^29^. During the first surgery, a 1 mm diameter craniotomy was made above the dorsal hippocampus on the right hemisphere (centred at AP, −2 mm; ML, +1.5 mm from bregma). An anchor screw was screwed into the skull on the contralateral hemisphere at approximately AP −1 mm and ML −2.5 mm from bregma. Then, 300 nl of AAV1-Syn-GCaMP6f was injected into dorsal CA1 of the hippocampus on the right hemisphere (AP, −2 mm; ML, +1.5 mm; DV, −1.2 mm). Virus was injected as described in the PSAM experiments above. After the pipette was removed, the mouse remained on the stereotaxic frame for 20 min to allow complete diffusion of the virus. After 20 min of diffusion, the cortex below the craniotomy was aspirated with a 27-gauge blunt syringe needle attached to a vacuum pump, while constantly being irrigated with cortex buffer. When the striations of the corpus callosum were visible, the 27-gauge needle was replaced with a 30-gauge needle for finer-tuned aspiration. Once most of corpus callosum was removed, bleeding was controlled using surgical foam (Surgifoam), and then a 1 mm diameter × 4 mm length GRIN lens (GRINTECH) was slowly lowered into the craniotomy. The lens was fixed with cyanoacrylate, and then dental acrylic was applied to cement the implant in place and cover the rest of the exposed skull. The top of the exposed lens was covered with Kwik-Sil (World Precision Instruments) to protect it and the Kwik-Sil was covered with dental cement. Then, 4 weeks later, the mice were again put under anaesthesia to attach the baseplate, visually guided by a Miniscope. The overlying dental cement was drilled off and the Kwik-Sil was removed to reveal the top of the lens. The Miniscope with an attached baseplate was lowered near the implanted lens and the field of view was monitored in real-time on a computer. The Miniscope was rotated until a well-exposed field of view was observed, at which point the baseplate was fixed to the implant with cyanoacrylate and dental cement. The mouse did not receive post-operative drugs after this surgery as it was not invasive. For inhibitory tagging experiments, the surgeries were performed as described above; however, they were separated into three surgeries rather than two: first, the virus injection was done and the mice had the incision sutured after the surgery. The lens implant procedure was done during a separate surgery 1–7 days later. Baseplating was done 1 month after viral injection during a third surgery.

For calcium imaging experiments with EEG/EMG implants (Fig. 5 and Extended Data Figs. 9 and 10), mice underwent three serial procedures spaced around 2 weeks apart. During the first surgery, mice had 300 nl of AAV1-Syn-GCaMP6f injected into dorsal CA1 as described above, but the incision was sutured after the surgery. Then, 2 weeks later during a second surgery, mice had their overlying cortex aspirated and a GRIN lens was implanted above the injection site, as above. During this surgery, a wireless telemetry probe (HD-X02, Data Science International) was also implanted with EEG and EMG wires. Two EMG wires were implanted into the left trapezius muscle. One EEG wire was implanted between the skull and dura mater above the dorsal hippocampus on the contralateral hemisphere to the GRIN lens (left hemisphere; AP, −2 mm; ML, −1.5 mm), and a reference EEG wire was implanted between the skull and the dura on the right hemisphere overlying the prefrontal cortex (AP, +1.75 mm; ML, −0.5 mm). Cyanoacrylate and dental cement fixed the GRIN lens, anchor screw and EEG wires in place. The telemetry probes were implanted during the second surgery rather than the first to minimize the time that the mice needed to live with the implant (because the mice sometimes reject the implant after long periods). During the third procedure, the mice were returned to implant the baseplate, as described above.

### Behavioural procedures

Before all of the experiments, the mice were handled for 1 min each day for at least 1 week. On at least four of those days, the mice were transported to the testing room and handled there. On the rest of the days, the mice were handled in the vivarium. In calcium imaging experiments, mice were handled and habituated for 2 weeks instead of 1, during which they were habituated to having the Miniscope attached and detached from their heads. To become accustomed to the weight of the Miniscope, they were placed in their home cage with the Miniscope attached for 5 min per day for at least 5 days.

In memory-linking behavioural experiments, mice were exposed to the neutral context for 10 min to explore. During aversive encoding, after a baseline period of 2 min, mice received three 2 s foot shocks of either amplitude 0.25 mA (low-shock) or 1.5 mA (high-shock), with an intershock interval of 1 min. Then, 30 s after the final shock, the mice were removed and returned to the vivarium. On the next 3 days, the mice were tested in the previously experienced aversive and neutral contexts, as well as a completely novel context that they had not been exposed to previously, for 5 min each. The features of the neutral and novel contexts were counter-balanced and were made up of different olfactory, auditory, lighting and tactile cues. The aversive context was always the same with distinct cues from the neutral and novel contexts. In the PSAM experiment (Extended Data Fig. 1l–p), the mice were tested in either the aversive, neutral or novel context. In the prospective versus retrospective memory-linking experiment (Fig. 1a–c), mice were tested in the aversive context first, and then half of the mice were tested in the neutral context and the other half in the novel context. In the low- versus high-shock experiments (Fig. 1d–g and Extended Data Figs. 1c–e and 9b,c), mice were tested in the aversive context first, followed by testing in the neutral and novel context counter-balanced; half of the mice received neutral recall and then novel-context exposure the next day, and the other half received novel-context exposure and then neutral recall. All testing was done in Med Associates chambers. Behavioural data were processed using the Med Associates software for measuring freezing. In experiments in which mice were tethered with a Miniscope, behavioural data were processed using our previously published open-source behavioural tracking pipeline, ezTrack^61^ v.1.2. In the prospective versus retrospective memory-linking temporal window experiments (Extended Data Fig. 1a,b), the aversive learning experience was distinct: mice explored for 2 min, then administered one 0.75 mA, 2 s foot shock and removed from the context 30 s after this shock.

In cocaine retrospective memory-linking experiments (Extended Data Fig. 2), mice were placed in the same contexts that were used in the above aversive memory-linking experiments (that is, Med Associates chambers). For cocaine–context pairings, mice were injected with cocaine (or saline as a control) and immediately placed in the conditioning context for 10 min. For encoding of the neutral context, mice were placed in the context for 10 min. Recall sessions were 5 min each. Behavioural data were processed using the Med Associates software for measuring locomotion.

### Drug injections

For PSAM experiments (Extended Data Fig. 1l–p), uPSEM-817 tartrate was made in a solution of 0.1 mg ml^−1^ in saline and injected intraperitoneally at a dose of 1 mg per kg (10 ml kg^−1^ injection volume). Previous studies have shown that PSAM4-GlyR (PSAM), an inhibitory ionotropic receptor with no endogenous ligand, binds with the injectable PSEM ligand to cause robust hyperpolarization in neurons^62^. Saline was used as a vehicle. The first injection was done as soon as the mice were brought back to the vivarium after aversive encoding (around 3 min after the end of aversive encoding). The next three injections were done every 3 h to cover a 12 h timespan of inhibition. For cocaine retrospective memory-linking experiments, mice were injected with 10 mg per kg (10 ml kg^−1^ injection volume) of cocaine dissolved in saline, or injected with saline as a control. For chemogenetic identification of inhibitory neuron experiments (Extended Data Figs. 5 and 6), clozapine *N*-oxide dihydrochloride (CNO) was made in a solution of 0.3 mg ml^−1^ in saline and injected intraperitoneally at a dose of 3 mg per kg (10 ml kg^−1^ injection volume). In Extended Data Fig. 5, all of the mice were injected with saline on the first day. On the second day, mice were injected with CNO or saline and, on the third day, mice were injected with saline or CNO, whichever solution they did not receive the day before.

### Calcium imaging Miniscope recordings

Open-source V4 Miniscopes (https://github.com/Aharoni-Lab/Miniscope-v4) were connected to a coaxial cable, which was connected to a Miniscope data acquisition board (DAQ) 3.3. The DAQ connected to a computer through USB3.0. Data were collected through the Miniscope QT Software v.1.11 (https://github.com/Aharoni-Lab/Miniscope-DAQ-QT-Software) at 30 fps. The Miniscopes were either assembled in-house or purchased from Open Ephys, and DAQ boards were purchased from Open Ephys.

When performing calcium imaging with concurrent behaviour in the Med Associates boxes, mice were brought into the testing room from the vivarium, taken out of their home cage and had the Miniscope attached. They were placed back into their home cage for 1 min. They were then removed from their home cage and placed into the testing chamber. To record calcium and behaviour, the Med Associates software sent a continuous TTL pulse to record from the Miniscope while the behaviour was concurrently tracked using Med Associates cameras. After the session was complete, the mice were immediately returned to their home cage, then the Miniscope was removed, and the mouse was returned to the vivarium. One mouse was brought to the testing room at a time.

For calcium imaging experiments without simultaneous EEG and EMG recordings, offline calcium imaging recordings were done in the mouse’s home cage for the 1 h after neutral encoding and after aversive encoding. During these recordings, mice were placed back into their home cage and the home cage was placed into a large rectangular and opaque storage bin to occlude distal cues, with a webcam (Logitech C920e or MiniCAM) overlying the home cage to track behaviour during the recording. Using the Miniscope QT Software with two devices connected (Miniscope and webcam), calcium imaging and behaviour were concurrently tracked. After the offline recording was complete, mice were removed from their home cage, the Miniscope was removed, they were returned to their home cage and returned to the vivarium immediately thereafter. The same procedure was undergone for the experiment in Extended Data Fig. 3. For calcium imaging experiments with simultaneous EEG and EMG recordings, mice lived in a custom-made home cage where offline recordings could take place. These home cages (Maze Engineers) were custom designed to accommodate mice wearing a Miniscope chronically for the duration of the experiment (about 2 weeks total). The water spout and food hopper were side-mounted and there was a slit along the top of the home cage so that the Miniscope coaxial cable could freely move. This home cage was placed on top of a receiver that would wirelessly receive EEG, EMG, temperature and locomotion telemetry data continuously throughout the experiment (HD-X02, Data Science International). Mice had a Miniscope attached on the first day and were allowed to wear it for an hour in their home cage to acclimatize to its weight, after which it was removed. On the second day, the Miniscope was attached and remained on for the duration of the experiment, for a total of 2 weeks. The Miniscope was connected to a lightweight coaxial cable (Cooner Wire) which connected to a low-torque passive commutator (Neurotek) to allow the mice to freely move around the home cage with minimal rotational force. After exposure to the neutral context during encoding, the mice were immediately returned to their home cage in the vivarium and the first calcium imaging recording began. The Miniscope DAQ was connected to an Arduino with a schedule set up to send a 10 min TTL pulse to record for 10 min, with a 20 min break in between, repeated 24 times. Thus, we sampled 4 h worth of calcium imaging data across 12 h. The telemetry probe recorded continuously for the duration of the experiment while the mouse was in its home cage in the vivarium.

### Sleep recordings and sleep scoring

The HD-X02 implants recorded EEG, EMG, temperature and locomotion continuously throughout the experiment at 100 Hz. After the experiment was completed, the data were run through an automatic custom-written algorithm to detect sleep states. First, the data were binned into 6 s epochs (to allow enough cycles of slow-wave oscillations). To separate sleep and wake states, the EMG data were fit with a Gaussian mixture model with two states, in which the lower state represented sleep and the higher state represented wake. To separate REM versus NREM periods, the EEG was band-pass filtered for theta (5–9 Hz) and delta (0.5–4 Hz) signals, and a ratio of theta to delta signal was calculated. A Gaussian mixture model was fit to this theta/delta ratio with two states, in which high theta/delta meant REM, while low theta/delta meant NREM. The algorithm was validated against manually scored data.

### Miniscope data processing and data alignment

To extract calcium transients from the calcium imaging data, we used our previously published open-source calcium imaging data processing pipeline, Minian^63^ v.1.2.1. In brief, videos were preprocessed for background fluorescence and sensor noise, and motion corrected. Putative cell bodies were then detected to feed into a constrained non-negative matrix factorization algorithm to decompose the three-dimensional video array into a three-dimensional array representing the spatial footprint of each cell, as well as a two-dimensional matrix representing the calcium transients of each cell. The calcium transients were then deconvolved to extract the estimated time of each calcium transient. Deconvolved calcium activities were analysed in these studies, except Extended Data Figs. 5 and 6, which used calcium traces. For calcium imaging experiments with EEG/EMG, data were processed as above; however, the videos were temporally downsampled by 2 (to 15 Hz). Cells recorded across sessions within a mouse were cross-registered using a previously published open-source cross-registration algorithm, CellReg, using the spatial correlations of nearby cells to determine whether highly correlated footprints close in space are likely to be the same cell across sessions^64^. For calcium imaging experiments with EEG/EMG, each offline recording was cross-registered with all the encoding and recall sessions, but not with the other offline sessions because cross-registering between all sessions would lead to too many conflicts and, therefore, to no cells cross-registered across all sessions.

To align calcium imaging data with behaviour, behaviour recordings were first aligned to an idealized template assuming a perfect sampling rate. This meant that if a recording session was 5 min, there should be 300 s × 30 fps = 9,000 frames (for a 30 Hz recording). All behaviour recordings were within four frames of this perfect template. Calcium recordings recorded with a much more variable and dynamic sampling rate. Then, for each behaviour frame, the closest calcium imaging frame was aligned to that frame, using the computer timestamp of that frame in milliseconds. No calcium imaging frame was reused more than twice. For calcium imaging experiments with EEG/EMG, each frame of calcium activity was aligned with the sleep state the mouse was in at that time. To do this, the computer time of each calcium frame was compared with the sleep states detected around the same time. If the calcium frame occurred during one of the 6 s sleep timeframes, that calcium frame was designated that sleep state; otherwise, if there were no sleep data during that time (due to data being dropped or low quality), it was designated no state and was excluded from sleep-state-specific analyses to account for any dropped frames in the telemetry data.

### General statistics and code/data availability

All analyses and statistics were performed using custom-written Python and R scripts. Code detailing all the analysis in this Article is available at GitHub (https://github.com/denisecailab/RetrospectiveMemoryLinkingAnalysis_2024). Calcium imaging data used in this Article is available through the Neurodata Without Borders framework to seamlessly share data across institutions upon reasonable request^65^. Statistical significance was assessed using two-tailed paired and unpaired *t*-tests, as well as one-way, two-way, or three-way analysis of variance, linear mixed-effects models or χ^2^ tests where appropriate. Significant effects or interactions were followed with post hoc testing with the use of contrasts or with Benjamini–Hochberg corrections for multiple comparisons. Significance levels were set to *α* = 0.05. Significance for comparisons is indicated by asterisks; *P ≤ 0.05, **P < 0.01, ***P < 0.001, ****P < 0.0001. Sample sizes were chosen on the basis of previous similar studies. Error bars and error bands always refer to the s.e.m., and bars and points with error bars always refer to the mean. The investigators were not blinded to behavioural testing in calcium imaging studies but were blinded to behavioural testing in all other experiments. Mice were randomly assigned to groups in all of the experiments.

### Ensemble reactivation analysis

To measure ensemble reactivation across the offline period (Extended Data Fig. 4f), for each mouse, the matrix of neural activity that was recorded during the offline session was *z*-scored along both axes (cells and time). Cells were then broken up into ensembles on the basis of whether they were previously observed to be active. Previously active cells were defined on the basis of whether they had a corresponding matched cell through CellReg. On offline 1 after neutral encoding, cells were either previously matched to an active cell during neutral encoding (neutral ensemble) or had no previously matched cell (remaining ensemble). On offline 2, cells had a matched cell only with neutral encoding and not aversive encoding (neutral ensemble), a matched cell with aversive encoding and not neutral encoding (aversive ensemble), a matched cell on both neutral encoding and aversive encoding (overlap ensemble), or no matched cell (remaining ensemble). For each ensemble, the activity of cells was averaged across cells, and then averaged across time for each time bin.

### Burst participation analysis

To measure population bursts (Figs. 2 and 3 and Extended Data Figs. 3 and 4), for each mouse, all cells that were recorded during that session were *z*-scored along the time dimension, such that each cell was normalized to its own activity. By doing this, no cell overly contributed to population bursts by having a very high amplitude event. Then, the mean population activity across the whole population was computed across the session and that one-dimensional trace was *z*-scored. Time periods when the mean population activity reached above a threshold of *z* = 2 were considered to be burst events. During each of these burst events, each cell was considered to have participated if its activity was above *z* = 2 during the event. For each ensemble (as defined in the previous section), the fraction of the ensemble that participated in each event was computed, and then this was averaged across all events. The average participation of each ensemble was compared across ensembles and across low- versus high-shock groups.

### Ensemble co-participation analysis

To measure ensemble co-participation during bursts (Figs. 4 and 5 and Extended Data Figs. 3, 6 and 8), bursts were defined on the basis of the *z*-scored mean population activity of the whole population. Then, for each burst event, the *z*-scored mean population activity was computed for the neutral ensemble and for the aversive ensemble (see the ‘Ensemble reactivation analysis’ section for ensemble definitions). For each population-level burst event, the ‘participation’ of the neutral ensemble or aversive ensemble was measured on the basis of whether the ensemble’s mean population activity was above the *z* = 2 threshold during the population level event. The burst events in which one ensemble participated without the other ensembles were considered independent participations. The burst events in which multiple ensembles simultaneously participated were considered co-participations. The fraction of burst events in which each ensemble independently participated and co-participated was computed. Then, the same computation was performed for all non-burst periods to examine how frequently the ensembles burst independently and coincidentally outside of burst events. In the calcium imaging experiment with EEG/EMG (Fig. 5), ensemble co-participation was defined above; however, as there were several offline recordings per mouse, each ensemble mean activity was computed for each offline session, and all the mean ensemble activities were concatenated to produce a pseudocontinuous time series of mean ensemble activities across the offline session. These mean activities were *z*-scored and then ensemble co-participation was computed separately for each sleep state.

### Time-lagged cross-correlation analysis

To measure cross-correlations (Extended Data Fig. 4k), mean ensemble activities were computed for the overlap, neutral and aversive ensembles (see the previous two sections). Each time series was then broken up into 120 s bins. The overlap ensemble was separately correlated with the neutral ensemble and the aversive ensemble bin by bin. For each time bin, cross-correlations were computed for lags up to a maximum of 5 frames (or ~160 ms). The maximum correlation was taken for each time bin, and the average correlation across time bins was computed. This led to, for each mouse, an average correlation between the overlap ensemble and the neutral ensemble, and an average correlation between the overlap ensemble and the aversive ensemble, across the offline period.

### Inhibitory neuron chemogenetic tagging (chemotagging)

To chemogenetically identify which neurons recorded with calcium imaging were inhibitory neurons (Extended Data Fig. 6), the calcium transients of cells during the 45 min CNO session were taken and normalized to have the range [0,1]. The number of prominent calcium peaks that each cell had from minutes 10–40 were computed and this was used to sort the cells from most to least responsive during this inhibitory tagging session (with cells with more peaks being more responsive and more likely putative GAD^+^ inhibitory neurons). These cells were cross-registered back to cells that were active during the previous offline 2 day (Extended Data Fig. 6b,c,h,j) to distinguish putative inhibitory neurons during that session. If a cell on offline 2 was not cross-registered with a cell on inhibitory tag day, that offline 2 cell was set to have 0 activity on inhibitory tag day, with the rationale that an hM3Dq^+^ cell would be likely to respond when administered with CNO. Offline cells were sorted on the basis of their responses on inhibitory tag day, with the most responsive cells being putative inhibitory neurons. Then, offline 2 cells were binned into groups on the basis of how responsive they were on inhibitory tag day (for example, top 20% of responsive cells) for downstream analyses. The same cross-registration was repeated with neutral and aversive encoding (Extended Data Fig. 6j) for decoding with putative inhibitory neurons. To compare putative inhibitory versus excitatory neurons (Extended Data Fig. 6b,c), the top 10% of most responsive cells on CNO day were used as the putative inhibitory neurons, with the rest of the population as putative non-inhibitory neurons. This is based on anatomical data estimating that inhibitory neurons make up about 10% of the neuronal population in the pyramidal layer on hippocampal CA1 (ref. ^45^).

### SVM analyses

To perform support vector machine (SVM) decoding to distinguish neutral from aversive encoding based on neural activity (Extended Data Fig. 7a), first only cells that were active during both encoding sessions were aligned and all other cells active during only one of the encoding sessions were excluded. As neutral encoding was longer than aversive encoding, neutral encoding activity was trimmed to the same length as aversive encoding. The activity vectors were concatenated and a random 50% of vectors were used to define the training set. A linear SVM was fit to the activity patterns and then tested for decoding accuracy on the held out 50% of data. This was repeated 50 times to produce a distribution of accuracies, from which the mean accuracy was extracted. For shuffle controls, the labels were randomly shuffled and the SVM was trained on the randomly shuffled labelled data. For SVM decoding in the inhibitory tagging experiment, first decoding was done as described above (Extended Data Fig. 6i). Second, cells active during both neutral and aversive encoding were extracted, as described above. These cells were sorted on the basis of how responsive they were on inhibitory tagging day (when they received CNO). The cells were broken up into fifths from most responsive to least responsive on inhibitory tagging day. Each 20% of cells was trained using an SVM as above (Extended Data Fig. 6j). This performance was compared with shuffled label controls for each fraction of cells.

### Population vector correlation analysis during encoding

To measure the similarity of population activity within and across neutral and aversive encoding, cells that were active during both neutral and aversive encoding were extracted (excluding any cells active only during one or the other), and the activities were concatenated across time. A population vector correlation matrix was computed to extract intrasession correlations (comparing every moment to every other moment within a session), as well as intersession correlations (comparing every moment within a session to all moments in the other session). The mean intrasession correlations were computed (intra-neutral and intra-aversive), as well as the intersession correlations (InterCorrs), and compared.

### Encoding-to-recall population vector correlation analysis

To measure correlations between encoding and recall activity patterns (Fig. 6b), first for each mouse, only cells that were active during both the encoding and recall session were included in the analysis and were aligned across the two sessions. For the encoding session, the mean population activity across the entire session was computed to produce one vector. Then, the recall session was broken up into 30 s bins and the mean population activity vector was computed for each bin. The encoding vector was correlated with each recall vector, as described previously^66^. We used Kendall’s tau correlations. Finally, the correlations across all of the recall bins were averaged to produce one average correlation between encoding and recall, for each mouse.

### Ensemble reactivation during neutral and novel recall

To measure reactivation of past encoding ensembles during recall (Fig. 6a and Extended Data Fig. 4q–s), for each mouse, cells active during neutral and novel recall were cross-registered with cells active during neutral encoding and not aversive encoding (neutral ensemble), aversive encoding and not neutral encoding (aversive ensemble), and during both neutral and aversive encoding (overlap ensemble). The fraction of recall cells that were cross-registered with each of these ensembles was then computed (for example, the fraction of neutral recall cells that were previously active during both neutral and aversive encoding—the overlap ensemble, measured the reactivation of the overlap ensemble during neutral recall). These values of ensemble reactivation are reported in Extended Data Fig. 4q–s for the reactivation of the neutral, aversive and overlap ensembles during neutral and novel recall. Then, for each mouse, the difference in this reactivation between neutral and novel recall was computed (neutral reactivation − novel reactivation) to create a reactivation index. A reactivation index of greater than 0 would indicate that an ensemble was more reactivated in neutral compared to novel recall. A value less than 0 would indicate that the ensemble was more reactivated during novel recall. These reactivation index scores are reported in Fig. 6a.

### Inclusion and ethics statement

All authors support inclusive, diverse and equitable research conduct. Eight authors self-identify as part of an under-represented group in biomedical research as defined by the NIH. Moreover, nine authors, including the senior author, are women. One or more authors received support from a program designed to increase diverse representation in science, including the NIH Diversity Supplement and Mount Sinai Scholar Award.

### Reporting summary

Further information on research design is available in the Nature Portfolio Reporting Summary linked to this article.

## Online content

Any methods, additional references, Nature Portfolio reporting summaries, source data, extended data, supplementary information, acknowledgements, peer review information; details of author contributions and competing interests; and statements of data and code availability are available at 10.1038/s41586-024-08168-4.

## Supplementary information

Reporting Summary

## Data Availability

The experimental data supporting the findings of this study are available at GitHub (https://github.com/denisecailab/RetrospectiveMemoryLinkingData_2024).
