# Supplementary material for: Offline ensemble co-reactivation links memories across days
Source: Nature. 2024 Nov 6;637(8044):145–55. doi: 10.1038/s41586-024-08168-4 (PMC11666460; doi:10.1038/s41586-024-08168-4)
Supplement: Supplementary file 1 — Reporting Summary [file 41586_2024_8168_MOESM1_ESM.pdf]

nature portfolio

Corresponding author(s): Denise J. Cai

Last updated by author(s): 9/20/2024

Reporting Summary

Nature Portfolio wishes to improve the reproducibility of the work that we publish. This form provides structure for consistency and transparency in reporting. For further information on Nature Portfolio policies, see our Editorial Policies and the Editorial Policy Checklist.

Statistics

For all statistical analyses, confirm that the following items are present in the figure legend, table legend, main text, or Methods section.

n/a	Confirmed	
☐	☒	The exact sample size (*n*) for each experimental group/condition, given as a discrete number and unit of measurement
☐	☒	A statement on whether measurements were taken from distinct samples or whether the same sample was measured repeatedly
☐	☒	The statistical test(s) used AND whether they are one- or two-sided<br>*Only common tests should be described solely by name; describe more complex techniques in the Methods section.*
☐	☒	A description of all covariates tested
☐	☒	A description of any assumptions or corrections, such as tests of normality and adjustment for multiple comparisons
☐	☒	A full description of the statistical parameters including central tendency (e.g. means) or other basic estimates (e.g. regression coefficient) AND variation (e.g. standard deviation) or associated estimates of uncertainty (e.g. confidence intervals)
☐	☒	For null hypothesis testing, the test statistic (e.g. *F*, *t*, *r*) with confidence intervals, effect sizes, degrees of freedom and *P* value noted<br>*Give P values as exact values whenever suitable.*
☒	☐	For Bayesian analysis, information on the choice of priors and Markov chain Monte Carlo settings
☐	☒	For hierarchical and complex designs, identification of the appropriate level for tests and full reporting of outcomes
☒	☐	Estimates of effect sizes (e.g. Cohen's *d*, Pearson's *r*), indicating how they were calculated

Our web collection on statistics for biologists contains articles on many of the points above.

Software and code

Policy information about availability of computer code

Data collection	Behavioral data were recorded with either Med Associates hardware and software (i.e., VideoFreeze) or with open-source Miniscope acquisition software version 1.11 (https://github.com/Aharoni-Lab/Miniscope-DAQ-QT-Software). Miniscope data were also recorded with the Miniscope acquisition software, using open-source V4 Miniscope hardware (https://github.com/Aharoni-Lab/Miniscope-v4). EEG/EMG data were recorded using telemetry probes and recording hardware from Data Sciences International (DSI).
Data analysis	Behavioral data were processed with Med Associates VideoFreeze software for experiments where mice were not tethered with a cable. For Miniscope experiments, behavioral data were processed using our previously published open-source software, ezTrack version 1.2 (https://github.com/denisecailab/ezTrack). Miniscope data were processed using our previously published open-source software, Minian version 1.2.1 (https://github.com/denisecailab/minian). Cells were tracked across days using the previously published open-source software CellReg (https://github.com/zivlab/CellReg). Behavioral and Miniscope data were analyzed using custom-written scripts in Python and R, which will be made available on https://github.com/denisecailab.

For manuscripts utilizing custom algorithms or software that are central to the research but not yet described in published literature, software must be made available to editors and reviewers. We strongly encourage code deposition in a community repository (e.g. GitHub). See the Nature Portfolio guidelines for submitting code & software for further information.

Data

Policy information about availability of data

All manuscripts must include a data availability statement. This statement should provide the following information, where applicable:

- Accession codes, unique identifiers, or web links for publicly available datasets
- A description of any restrictions on data availability
- For clinical datasets or third party data, please ensure that the statement adheres to our policy

All data in this manuscript will become publicly available using the Neurodata Without Borders format for ease of use by the research community. Analysis code used in this manuscript will become fully available on https://github.com/denisecailab.

Research involving human participants, their data, or biological material

Policy information about studies with human participants or human data. See also policy information about sex, gender (identity/presentation), and sexual orientation and race, ethnicity and racism.

Reporting on sex and gender	n/a
Reporting on race, ethnicity, or other socially relevant groupings	n/a
Population characteristics	n/a
Recruitment	n/a
Ethics oversight	n/a

Note that full information on the approval of the study protocol must also be provided in the manuscript.

Field-specific reporting

Please select the one below that is the best fit for your research. If you are not sure, read the appropriate sections before making your selection.

☒ Life sciences ☐ Behavioural & social sciences ☐ Ecological, evolutionary & environmental sciences

For a reference copy of the document with all sections, see nature.com/documents/nr-reporting-summary-flat.pdf

Life sciences study design

All studies must disclose on these points even when the disclosure is negative.

Sample size	Sample sizes were determined from prior behavioral and calcium imaging studies on memory-linking (Cai et al., Nature, 2016).
Data exclusions	No data were excluded from behavioral experiments except for one calcium imaging mouse which had a seizure during the experiment, and four mice from the cocaine experiment in Extended Figure 1A-C because of an experimenter mistake when transporting mice to the testing room. 2 mice were excluded from calcium imaging due to overexpression of the virus determined by spreading depression. The calcium imaging from these 3 mice were never processed and excluded prior to data analysis. For analyses that required cross-registration of cells across sessions, 3 mice were excluded because of an inability to register cells across sessions.
Replication	The retrospective memory-linking behavioral result was replicated in Figure 1C, Figure 1F, Extended Figure 1B, Extended Figure 1E, Extended Figure 1H, Extended Figure 1K, and Extended Figure 9C, and replicated by different experimenters. The calcium imaging experiment in the main figures (Fig 1-4; Low vs High Shock retrospective memory-linking) was run across two separate cohorts and collapsed. The ensemble co-bursting result was replicated in Figure 4E, Figure 5C, and Extended Figure 6F. The cocaine memory-linking result in Extended Figure 2I was replicated across two cohorts and collapsed.
Randomization	All mice were randomly assigned to groups in all experiments.
Blinding	Experimenters were blinded to experimental conditions for all experiments except for the calcium imaging experiments as calcium imaging required running one animal at a time. All analyses were automated and performed blinded to groups, including calcium imaging experiments.

Reporting for specific materials, systems and methods

We require information from authors about some types of materials, experimental systems and methods used in many studies. Here, indicate whether each material, system or method listed is relevant to your study. If you are not sure if a list item applies to your research, read the appropriate section before selecting a response.

Materials & experimental systems

n/a	Involved in the study
☒	☐ Antibodies
☒	☐ Eukaryotic cell lines
☒	☐ Palaeontology and archaeology
☐	☒ Animals and other organisms
☒	☐ Clinical data
☒	☐ Dual use research of concern
☒	☐ Plants

Methods

n/a	Involved in the study
☒	☐ ChIP-seq
☒	☐ Flow cytometry
☒	☐ MRI-based neuroimaging

Animals and other research organisms

Policy information about studies involving animals; ARRIVE guidelines recommended for reporting animal research, and Sex and Gender in Research

Laboratory animals	C57/BL6J mice in every experiment except Extended Figures 5 and 6 in which Gad2-cre mice were used. Mice were 12-18 weeks of age when behavior was conducted. Mice were housed on a 12:12 hour light/dark cycle where lights were on during the day. Average ambient temperature was 73F and the humidity ranged from 30-65%.
Wild animals	This study did not use wild animals.
Reporting on sex	Only male mice were used in this study. Previous studies did not find differences in memory-linking across sexes (Cai et al., Nature, 2016).
Field-collected samples	This study did not involve field samples.
Ethics oversight	All experimental procedures were approved by the Icahn School of Medicine at Mount Sinai's IACUC.

Note that full information on the approval of the study protocol must also be provided in the manuscript.

Plants

Seed stocks	n/a
Novel plant genotypes	n/a
Authentication	n/a
